# The complete mitochondrial genomes of sixteen ardeid birds revealing the evolutionary process of the gene rearrangements

**DOI:** 10.1186/1471-2164-15-573

**Published:** 2014-07-08

**Authors:** Xiaoping Zhou, Qingxian Lin, Wenzhen Fang, Xiaolin Chen

**Affiliations:** Key Laboratory of Ministry of Education for Coast and Wetland Ecosystems, College of the Environment and Ecology, Xiamen University, Xiamen, 361102 People’s Republic of China

**Keywords:** Mitochondrial genome, Gene rearrangement, Concerted evolution, Phylogeny, Ardeidae

## Abstract

**Background:**

The animal mitochondrial genome is generally considered to be under selection for both compactness and gene order conservation. As more mitochondrial genomes are sequenced, mitochondrial duplications and gene rearrangements have been frequently identified among diverse animal groups. Although several mechanisms of gene rearrangement have been proposed thus far, more observational evidence from major taxa is needed to validate specific mechanisms. In the current study, the complete mitochondrial DNA of sixteen bird species from the family Ardeidae was sequenced and the evolution of mitochondrial gene rearrangements was investigated. The mitochondrial genomes were then used to review the phylogenies of these ardeid birds.

**Results:**

The complete mitochondrial genome sequences of the sixteen ardeid birds exhibited four distinct mitochondrial gene orders in which two of them, named as “duplicate tRNA^Glu^–CR” and “duplicate tRNA^Thr^–tRNA^Pro^ and CR”, were newly discovered. These gene rearrangements arose from an evolutionary process consistent with the tandem duplication - random loss model (TDRL). Additionally, duplications in these gene orders were near identical in nucleotide sequences within each individual, suggesting that they evolved in concert. Phylogenetic analyses of the sixteen ardeid species supported the idea that *Ardea ibis*, *Ardea modesta* and *Ardea intermedia* should be classified as genus *Ardea*, and *Ixobrychus flavicollis* as genus *Ixobrychus*, and indicated that within the subfamily Ardeinae, *Nycticorax nycticorax* is closely related to genus *Egretta* and that *Ardeola bacchus* and *Butorides striatus* are closely related to the genus *Ardea*.

**Conclusions:**

The duplicate tRNAThr–CR gene order is found in most ardeid lineages, suggesting this gene order is the ancestral pattern within these birds and persisted in most lineages via concerted evolution. In two independent lineages, when the concerted evolution stopped in some subsections due to the accumulation of numerous substitutions and deletions, the duplicate tRNAThr–CR gene order was transformed into three other gene orders. The phylogenetic trees produced from concatenated rRNA and protein coding genes have high support values in most nodes, indicating that the mitochondrial genome sequences are promising markers for resolving the phylogenetic issues of ardeid birds when more taxa are added.

**Electronic supplementary material:**

The online version of this article (doi:10.1186/1471-2164-15-573) contains supplementary material, which is available to authorized users.

## Background

Five distinct mitochondrial (mt) gene orders have been described in birds since the complete mt genome of the domestic chicken (*Gallus gallus*) was presented [1–17]. There are differences among the various copies and arrangements of the control region (CR) and flanking genes among these five mt gene orders, which were assumed to have resulted from the evolutionary process, tandem duplication and random loss (TDRL). Gibb et al. [8] introduced a naming system of avian mt gene orders according to these variations, known as ancestral avian, remnant CR (2), duplicate CR and duplicate tRNA^Thr^–CR. These were initially identified in the chicken [1], falcon [2], *Amazona* parrot [5] and albatross [7]. In ancestral avian gene order, the gene content and arrangement in the region located between cytochrome *b* (Cytb) and tRNA^Phe^ is Cytb, tRNA^Thr^, tRNA^Pro^, ND6, tRNA^Glu^ and CR. These genes and the CR are tandem duplicated in the duplicate tRNA^Thr^–CR gene order, but the second Cytb is only a partial copy (p-Cytb). In comparison with the duplicate tRNA^Thr^–CR gene order, the first tRNA^Pro^, ND6 and tRNA^Glu^ and the second tRNA^Thr^ are lost or reduced in the duplicate CR and remnant CR (2) gene orders. Additionally, there is degeneration of the second CR in the remnant CR (2) gene order. Besides these four orders, Verkuil et al. [13] identified a novel mt gene order in the ruff, which differs from the duplicate tRNA^Thr^–CR gene order in the degeneration of the second tRNA^Thr^.

In TDRL model, usually one copy of the duplicated genes or CRs loses function and degrades or is eventually deleted [18]. However, in birds it is common that the duplications have persisted for a long evolutionary time, where both copies of the duplicated genes or CRs are functional and have high sequence similarity. Such situation has generally been thought to be due to concerted evolution and multiple mechanisms accounting for concerted evolution were postulated [5, 7, 8, 10, 12–15, 19, 20].

The family Ardeidae is a group of large wading birds that is distributed across most parts of the world. According to a recent classification by Kushlan and Hancock [21], this family consists of five subfamilies, 17 genera and 62 species. Recent avian molecular phylogenies based on a large set of nuclear data [22] and complete mtDNA [17] have united the Ardeidae into a large taxonomic group that contains Pelecaniformes and Ciconiiformes. The duplicate tRNA^Thr^–CR gene order is taxonomically widespread within this group [7, 11, 12, 14, 17], suggesting that this mitochondrial gene order might be a general pattern in these related birds. However, the recently published complete mitochondrial DNA (mtDNA) sequences of four ardeid species indicate that their gene orders are similar to the ancestral gene order [8, 23, 24].

In the current study, the complete mtDNA of 16 ardeid species was sequenced in order to explore the occurrence of derived, rather than ancestral avian, mt gene orders in ardeid mt genomes and to investigate the evolution of mt gene rearrangements in the family Ardeidae. The phylogenetic relationships within the surveyed ardeid species were also reviewed using the concatenated nucleotide sequences of two rRNA and 12 protein coding genes.

## Methods

### Sample collection

Tissue samples were collected from sixteen ardeid species (Table 1). The nomenclature used for ardeid species in this study is the taxonomic system proposed by Kushlan and Hancock [21]. The muscle or liver tissue samples used in this study were collected from dead birds found in the wild during field investigations and stored in alcohol until DNA extraction. The Administration Center for Wildlife Conservation in Fujian Province (FJWCA-1208) approved the current research and all procedures involving animal tissue collection in the wild. The scientific license for access to the study site was issued by the Administration Department of Xiamen Egret Natural Reserve (XMENR-1005).

**Table 1:** **Ardeid species sampled in this study**

Common name	Scientific name	Collection location
**Ardeinae**		
Grey Heron	*Ardea cinerea*	Xiamen, Fujian
Purple Heron	*Ardea purpurea*	Changchun, Jilin
Eastern Great Egret	*Ardea modesta*	Xiamen, Fujian
Intermediate Egret	*Ardea intermedia*	Nanjing, Jiangsu
Cattle Egret	*Ardea ibis*	Xiamen, Fujian
Striated Heron	*Butorides striatus*	Nanjing, Jiangsu
Chinese Pond Heron	*Ardeola bacchus*	Xiamen, Fujian
Little Egret	*Egretta garzetta*	Xiamen, Fujian
Chinese Egret	*Egretta eulophotes*	Zhangzhou, Fujian
Eastern Reef heron	*Egretta sacra*	Zhangzhou, Fujian
Black-crowned Night Heron	*Nycticorax nycticorax*	Xiamen, Fujian
**Botaurinae**		
Eurasian Bittern	*Botaurus stellaris*	Changchun, Jilin
Yellow Bittern	*Ixobrychus sinensis*	Haikou, Hainan
Schrenck’s Bittern	*Ixobrychus eurhythmus*	Haikou, Hainan
Cinnamon Bittern	*Ixobrychus cinnamomeus*	Haikou, Hainan
Black Bittern	*Ixobrychus flavicollis*	Longyan, Fujian

### DNA extraction, PCR amplification and sequencing

Whole genomic DNA was extracted using the DNeasy Blood and Tissue Kit (Qiagen) following the manufacturer’s protocol.

To minimize the possibility of obtaining nuclear copies of mt genes, the entire mt genome was first amplified in two long overlapping fragments via long and accurate polymerase chain reaction (LA PCR) using primer pairs of LACOIF and LA16SR, LA12SF and LACOIIIR (Additional file 1) [25] and the Premix LA Taq kit version 2.0 (Takara, China). LA PCR was carried out in a Veriti 96 well thermocycler (Applied Biosystems). The final reaction volume of 50 μL contained 1 μL (about 100 ng) of genomic DNA, 2.5 U LA Taq Polymerase, 1× LA PCR buffer II, 0.4 mM of each dNTP, and 0.1 μM of each primer. PCR cycling conditions were as follows: 1 min denaturation at 94°C, followed by 30 cycles of 15 s at 94°C, 10 min at 68°C, with a final extension at 72°C for 10 min. Each PCR yielded a single band, which was detected in a 0.8% agarose gel. The band was extracted and purified using a DNA Gel Extraction Kit (Axygen).

The purified PCR products were used as templates for nested PCR amplification with 19 primer sets (Additional file 1) [23, 26], using Premix Taq® kit version 2.0 (Takara). The strategy employed to amplify the region between Cytb and 12S rRNA is shown in Additional file 2. All nested PCR reactions were carried out in a final reaction volume of 50 μL containing 1 μL of template DNA, 1.25 U Taq Polymerase, 1× PCR buffer, 1.5 mM MgCl_2_, 0.2 mM of each dNTP, 0.1 μM of each primer. PCR cycling was carried out in a Veriti 96 well thermocycler (Applied Biosystems). PCR condictions were as follows: 3 min denaturation at 94°C, followed by 30 cycles of 15 s at 94°C, 15 s at 52°C, 2 min at 72°C, with a final extension at 72°C for 7 min. The amplification products were separated on a 1% agarose gel and DNA fragments from the individual bands were purified, ligated into pMD 18-T vector (TaKaRa) and transformed into *Escherichia coli* DH5α. Positive clones were selected to sequence in both directions and each sequenced twice independently. Sequencing was performed on an ABI PRISM 3730 automatic sequencer (Applied Biosystems) using universal M13 sequencing primers and BigDye version 3.1 (Applied Biosystems).

### Gene identification and genome analysis

Contiguous sequences that were obtained were assembled using the DNAMAN version 5.2 (Lynnon Biosoft). Most tRNA genes were identified using tRNAscan-SE 1.21 (http://lowelab.ucsc.edu/tRNAscan-SE/) with the vertebrate mitochondrial genetic code and ‘mito/chloroplast’ source. The tRNA^Ser^ (AGY), which not found by the tRNA-SE1.21, and protein coding genes were identified by homology alignments with the corresponding known sequences of *E. novaehollandiae*, *E. eulophotes*, *N. nycticorax*, and *I. cinnamomeus* [GenBank: DQ780878, EU072995, JN018412, HQ690247, respectively] using Clustal × 2.0 [27]. The boundaries of ribosomal RNA genes (rRNA) and CR^2^ were inferred from boundaries of flanking genes under the assumption that there are neither intergenic spacers nor overlaps. The boundary of the 5’ end of CR^1^ was inferred from the flanking tRNA^Glu^ or sequence homology to CR^2^ if the flanking tRNA^Glu^ was absent. The boundary of the 3’ end of CR^1^ was inferred from the sequence homology to CR^2^. The pair-wise alignments between putative pseudogenes and corresponding functional copy within an individual were performed in the LALIGN query program using default parameters (http://fasta.bioch.virginia.edu/fasta_www2/fasta_www.cgi?rm=lplalign) [28].

### Phylogenetic analysis

The following mtDNA from Threskiornithinae were retrieved from GenBank for phylogenetic analysis: *Nipponia nippon* [GenBank: NC_008132], *Platalea minor* [GenBank: NC_010962], *P. leucorodia* [GenBank: NC_012772] and *Threskiornis aethiopicus* [GenBank: NC_013146], which served as outgroups.

Nucleotide sequences of the two rRNAs and 12 protein coding genes encoded on heavy strands (excluding the stop codon) were used for phylogenetic analysis. Alignments were first built for all 14 genes individually using Clustal × 2.0 with default settings and then concatenated into a single alignment. Gaps and ambiguous areas in the alignments of the two rRNA genes were excluded using Gblocks 0.91b [29] with default parameters. The final alignment encompassed 20 taxa and 13, 184 nucleotide sites, of which 3, 882 sites were parsimony informative.

The concatenated alignment dataset was partitioned for phylogenetic analysis. First, the dataset was partitioned by 12 s rRNA, 16 s rRNA and each codon position of each protein coding gene. PartitionFinder v1.1.1 [30] was then used to determine the best partitioning scheme and nucleotide substitution models according to the Bayesian information criterion (BIC) and “greedy” algorithm with branch lengths estimated as “unlinked”. The analysis suggested splitting the dataset into three partitions as the best partitioning scheme: (1) 12 s rRNA  + 16 s rRNA + the first codon position of the 12 protein coding genes + the second codon position of ATP8, (2) the second codon position of the 11 protein coding genes, and (3) the third codon position of the 12 protein coding genes. The proposed best-fit nucleotide substitution model for partitions (1) and (3) was a general time-reversible model with gamma distribution substitution rates and proportion of invariable sites (GTR + I + G), and for partition (2) was the Hasegawa-Kishino-Yano model with a proportion of invariant sites and gamma distributed rate variation (HKY + I + G). These results were implemented in the following phylogenetic analyses.

Phylogenetic analyses were conducted using the Bayesian inference (BI) and maximum likelihood (ML) methods available on the CIPRES Science Gateway v3.3 [31]. The BI analysis was performed using MrBayes v3.1.2 [32] on XSEDE . Two sets of four chains were allowed to run simultaneously for 10,000,000 generations and each set was sampled every 1000 generations. Convergence and mixing of the chain of each analysis was evaluated using Tracer v1.6.1 (http://tree.bio.ed.ac.uk/software/tracer) to check that the ESS values were all superior to 200. A consensus tree was then calculated after omitting the first 25% trees as burn-in. The ML analysis was performed using RAxML-HPC2 on XSEDE v8.0.9 [33] and 1000 bootstraps were used to estimate the node reliability. Additionally, the GTR + G + I model was used for all three partitions, since the HKY + I + G model is not implemented in RAxML.

## Results

### Gene content and order of CR and flanking genes

**Figure 1:**
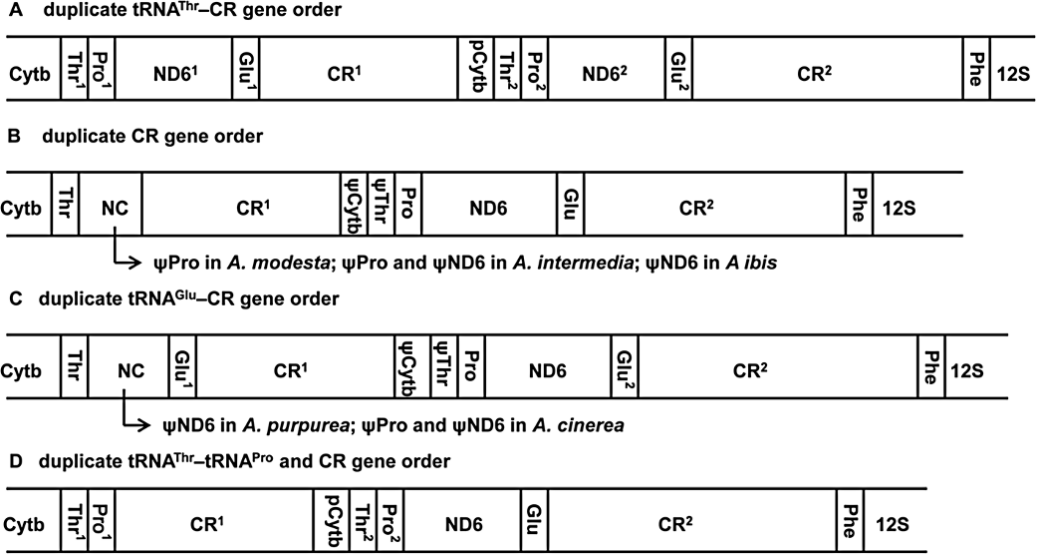
**Mitochondrial gene orders in ardeid birds.** Only the region between the Cytb and 12S rRNA is shown. **A**: duplicate tRNA^Thr^–CR gene order; **B**: duplicate CR gene order; **C**: duplicate tRNA^Glu^–CR gene order; **D**: duplicate tRNA^Thr^–tRNA^Pro^ and CR gene order. The gene order names follow the description by Gibb et al. [8]. pCytb: partial Cytb. ψ: pseudo gene.

**Figure 2:**
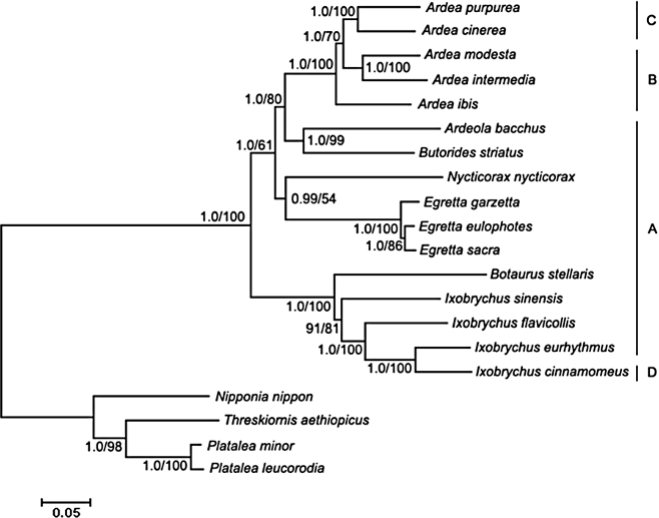
**Phylogenetic relationships among the sequenced ardeid birds.** Numbers at the nodes are Bayesian posterior probabilities (left) and ML bootstrap percentages (right). The letters to the right of the species name refers to gene orders shown in Figure 1.

In this study, the complete mitochondrial DNA sequences were obtained for sixteen ardeid birds, which had been deposited in NCBI GenBank under accession numbers KJ190944 to KJ190959. The size of deposited sequences varied substantially, from 18640 bp (*I. cinnamomeus*) to 20350 bp (*B. stellaris*). In the region between Cytb and tRNA^Phe^, all sixteen mitochondrial genomes contained two CRs, but the number of tRNA^Thr^, tRNA^Pro^, tRNA^Glu^ and ND6 copies varied (Figure 1). Four gene order types were exhibited in the sixteen genomes due to these variations: 1) in ten species, including *E. eulophotes*, *E. sacra*, *E. garzetta*, *B. striatus*, *A. bacchus*, *N. nycticorax*, *B. stellaris*, *I. sinensis*, *I. eurhythmus*, and *I. flavicollis*, the gene order was similar to the duplicate tRNA^Thr^–CR gene order and was characterized by a tandem duplicated region that spanned the last part of Cytb to the CR (Figure 1A); 2) in *A. modesta*, *A. intermedia* and *A. ibis*, the gene order was similar to the duplicate CR gene order, where tRNA^Pro1^, ND6^1^ and tRNA^Glu1^ as well as the partial Cytb and tRNA^Thr2^ were replaced by putative pseudogenes with respect to the duplicate tRNA^Thr^–CR gene order (Figure 1B); 3) in *A. cinerea* and *A. purpurea*, the gene order was similar to the duplicate CR gene order with exception of two copies of tRNA^Glu^ (Figure 1C); 4) in *I. cinnamomeus*, the gene order differed from the duplicate tRNA^Thr^–CR gene order in that tRNA^Pro1^ was immediately followed by CR^1^, which began with a poly C stretch (Figure 1D). The latter two gene orders are novel for birds, and for the sake of congruence with Gibb et al. [8], they were named as a duplicate tRNA^Glu^–CR gene order and a duplicate tRNA^Thr^–tRNA^Pro^ and CR gene order, respectively.

### Comparison of the duplicated regions

There were duplicated CRs in the four gene orders, both of which were deemed as complete since they both contained CR-specific conserved sequence blocks previously described in other avian research (poly C stretch, TAS motif, F box, E box, D box, C box, B box, bird similarity box, and CSB1) [34–38] (Additional file 3). Sequence alignments indicated that the sequences near the poly C stretch were usually highly similar between the duplicated CRs within each individual, while the middle parts of domain I were significantly divergent except in *I. flavicollis* and *B. stellaris*. After this, approximately 200 bp (150 bp in *A. bacchus* and *I. cinnamomeus*) at the 3’ end of domain I and all of domain II (defined from the start of the F box to the start of the CSB1) continued nearly identical between CR^1^ and CR^2^. CR^1^ domain III also coincided with the 5’ end of CR^2^ domain III. After this region, there were several hundred additional nucleotides, composed primarily of complicated CA-rich repetitive sequences, in CR^2^. In seven species, another repetitive sequence with a remarkably long motif was found in domain III of both CR^1^ and CR^2^. The sizes of the motifs were 93 bp in *A ibis*, 92 bp in *I. sinensis*, 91 bp in *B. stellaris* and *A. modesta*, and 81 bp in the three *Egretta* species. In the surveyed specimens, the minimum number of repeats was 3.2 in CRs of *A. modesta*, and the maximum number was 10 in CR^2^ of *B. stellaris* although heteroplasmy was present in this case (Additional file 3).

The other duplicated regions in the duplicate tRNA^Thr^–CR gene order, beginning from the 3’ end of Cytb through tRNA^Glu^ (duplicated Cytb section vary between 81 and 113 bp in length among the species) were also identical or nearly identical to each other within each individual. However, CR^1^ and the p-Cytb were separated by a small non-coding spacer (varying in length between 13 and 40 bp among species), which was not present in CR^2^ or Cytb. In *I. cinnamomeus* that had the duplicate tRNA^Thr^–tRNA^Pro^ and CR gene order, this intervening spacer was 26 bp in length. Additionally, in this individual, the duplicated region beginning from the last 88 bp of Cytb through tRNA^Pro^, differed by one mismatch base.

In the duplicate tRNA^Glu^–CR gene order, the sizable non-coding section (NC1, 217 bp in *A. cinerea* and 220 bp in *A. purpurea*), located between tRNA^Thr^ and tRNA^Glu1^, was presumed to be pseudogenes, which corresponded to remnants of the degenerated tRNA^Pro^ and ND6. Moreover, another sizable non-coding section (NC2, 166 bp), located between CR^1^ and tRNA^Pro^, was presumed to be pseudogenes, which corresponded to remnants of the degenerated Cytb and tRNA^Thr^. in order to verify the reliability of this mechanism, the degree of similarity between the NCs and corresponding functional genes was examined by pair-wise alignments within individuals of respective species. In *A. cinerea*, there was a 132 bp portion in NC1that had 68.8% identity with tRNA^Pro^ and ND6, a 42 bp portion in NC1 that had 85.7% identity with ND6, and a 142 bp portion in NC2 that had 72.3% identity with Cytb and tRNA^Thr^. In *A. purpurea*, there was a 194 bp portion in NC1 that had 63% identity with ND6 which especially exhibits 81.8% identity over the last 66 bp, and a 141 bp portion in NC2 that had 68% identity with Cytb and tRNA^Thr^ (Additional file 4).

Similarly, in the duplicate tRNA^Glu^–CR gene order, tRNA^Pro^-like or ND6-like subsections were found in NC1 located between tRNA^Thr^ and CR1, and Cytb-like and tRNA^Thr^-like subsections were found in NC2 (Additional file 4). Specifically, in *A. modesta*, a 76 bp portion in NC1 has 76.3% identity with tRNA^Pro^ and a 132 bp portion in NC2 has 66.0% identity with Cytb and tRNA^Thr^. In *A. intermedia*, a 225 bp portion in NC1 has 63.6% identity with the tRNA^Pro^ and ND6, and a 122 bp portion in NC2 has 63.6% identity with Cytb and tRNA^Thr^. In *A. ibis*, a 155 bp portion in NC1 has 58.8% identity with the ND6 and a 114 bp portion in NC2 has 62.6% identity with Cytb and tRNA^Thr^.

### Phylogenetic analysis

The BI and ML trees had similar topologies and most nodes were supported by high posterior probabilities (PP) and bootstrap percentages (BP) (Figure 2). In the trees, the 16 ardeid birds were first split into two monophyletic clades corresponding to the subfamilies Botaurinae and Ardeinae. Within the Botaurinae clade, the *Ixobrychus* birds formed a monophyletic clade with *B. stellaris* as the basal sister group. Within the Ardeinae clade, *N. nycticorax* was closely related to *Egretta*; *A. bacchus* and *B. striatus* formed a group which was closely related to *Ardea*. Within *Ardea*, the *A. ibis* was basal and *A. modesta* was sister to *A. intermedia*.

## Discussion

### Phylogenetic relationships among the surveyed ardeid birds

Among the surveyed ardeid birds, the major phylogenetic controversies have been related to the classifications of the *A. ibis*, *A. modesta*, *A. intermedia* and *I. flavicollis* and to the genus relationships within Ardeinae [39–45]. This study confirmed Sheldon’s postulate based on DNA-DNA hybridization data [44] that *A. ibis*, *A. modesta* and *A. intermedia* are members of the *Ardea* genus as they are more closely related to two typical *Ardea* species. *I. flavicollis* is generally received as a member of *Ixobrychus* [21, 43], but has sometimes been put in a monotypic genus *Dupetor* [41]. The present results indicate that *I. flavicollis* should be included in the genus *Ixobrychus* since it nested in this group. Moreover, the relationship between the genera, *Ardeola* and *Butorides*, was found to be the closest since *A. bacchus* and *B. striatus* were the first to form a group with high support (PP = 1 and BP = 99). This is consistent with the result based on osteological characters reported by Payne and Risley [43]. The group of *Ardeola* and *Butorides* was more closely related to the genus *Ardea*, which is in accord with the postulate of Kushlan and Hancock [21]. The closest relationship between the genera *Nycticorax* and *Egretta*, a newly found in current study, was strongly supported by BI analysis (PP = 0.99), but only weakly supported by ML analysis (BP = 54) and it could not be ruled out as a result of the long branch attraction [46].

In brief, our results were broadly consistent with previously phylogenetic studies based on DNA hybridization [44], Cytb [45] and vocalization [40]. These have been so far the only available datasets to estimate ardeid bird phylogeny. Although these methods are only rarely applied in current phylogenetic analyses, they have yielded highly congruent results [42]. Furthermore, in this study, most nodes in the phylogenetic trees had high support values, especially in the BI tree. The complete mitochondrial genomes may be the promising markers for resolving ardeid phylogenetic relationships at low taxonomic levels. In order to break up some long branches, avoid long-branch attraction and improve phylogenetic accuracy, it will be necessary to add more taxa, particularly the inclusion of certain key groups such as genera *Nyctanassa*, *Gorsachius*, and *Syrigma*, and more species in *Nycticorax* and *Ardeola*. Moreover, because the entire mitochondrial genome is inherited as a single locus, it would be highly desirable to include further unlinked nuclear makers into future phylogenetic analyses on ardeid birds.

### Ardeid mitochondrial gene orders

The complete mtDNAs of sixteen ardeid species exhibited four gene order types, all of which contained duplications. In light of the current results, we presume that mt gene orders with duplications were common in all ardeid birds, or at least in Ardeinae and Botaurinae subfamilies. The ancestral avian gene order previously found in *E. eulophotes* [23], *N. nycticorax*, and *I. cinnamomeus* [24] have been proven to be misidentifications in present study. Although the mt gene order of *E. novaehollandiae* [8] was not reexamined here, it is very likely that the ancestral avian gene order found in this species was also a misidentification. Indeed, in the sequencing of *E. novaehollandiae* mtDNA, Gibb et al. found indications of a potential duplication, but could not extensively test to completely exclude co-amplification of nuclear copies due to poor DNA quality [17].

Two of the four different mt gene orders in ardeid birds, the duplicate tRNA^Glu^–CR and duplicate tRNA^Thr^–tRNA^Pro^ and CR, were newly discovered, whereas the other two had been found previously in other avian species. The duplicate tRNA^Thr^–CR gene order had previously been reported in Philippine hornbills [15], albatrosses [7, 12], the black-faced spoonbill [11], and several birds of “core” pelecaniforms [14]. The p-Cytb was significantly shorter in ardeid birds compared to these birds, which implies that the extant length of the p-Cytb in ardeid birds was not the initial duplicated piece, but resulted from degeneration of the 5’ end of p-Cytb. Such a case has been reported in *Thalassarche* albatrosses [7], where a degenerate partial copy of Cytb (d-Cytb, retaining 70% sequence similarity to the functional Cytb) was found to precede the p-Cytb (retaining 100% identity to the 3’ end portion in the functional copy). The duplicate CR gene order has previously been reported for parrots and cockatoos [5, 16], osprey [8], ivory-billed aracari [8], blackcap and reed warbler [9]. In these cases, the p-Cytb and the pseudo copies of tRNA^Pro1^, ND6^1^, tRNA^Glu1^ and tRNA^Thr2^ are usually decayed beyond recognition. The only exception was *Amazona* parrots [5], in which identifiable pseudo ND6 and tRNA^Glu^ were still present. In current study, many recognizable pseudogenes were present and had more than 60% sequence identity with the portions of corresponding functional copies, making the extent of the duplication more easily defined and providing visible evidence that one copy of the duplicated genes was in the process of being eliminated.

### Evolution of ardeid mt gene rearrangements

The existence of tandem duplications and pseudogenes in ardeid mt genomes made it clear that the mt gene rearrangements arose from a process consistent with the TDRL model. The duplicate tRNA^Thr^–CR gene order was derived from the ancestral avian gene order by a tandem duplication of the region spanning from Cytb (or just the last part) to CR, followed by degeneration of the 3’ portion of the CR^1^ and 5’ portion of the Cytb. The other three gene orders were derived from the duplicate tRNA^Thr^–CR gene order by further degenerations or deletions of one copy of specific duplicated genes.

Since the duplicate tRNAThr–CR gene order was shared by most lineages (Figure 2) and the p-Cytbs were nearly identical in length not only among closely related but also distantly related species, it was most parsimonious that the origin of the duplicate tRNAThr–CR gene order preceded before the divergence of these ardeid birds. When this hypothesis was accepted, it is easier to explain the variation in ardeid gene orders, since the other three gene orders could have been more easily derived from the duplicate tRNAThr–CR gene order than from the ancestral avian gene order via accumulation of substitutions and deletions. Alternatively, although tandem duplication might arise multiple times within ardeid birds, this was less likely because the same replication errors, same tandem duplications and same degenerations would have had to repeatedly occur in each species [47].

In *I. cinnamomeus*, complete deletion of ND6^1^ and tRNA^Glu1^ as well as the 5’ end portion of CR^1^ (the portion upstream of C-stretch in CR^2^ is absent in CR^1^) had independently occurred, transforming the duplicate tRNA^Thr^–CR gene order into the duplicate tRNA^Thr^–tRNA^Pro^ and CR gene order. In the lineage leading to the five *Ardea* species, the degenerations or deletions of tRNA^Pro1^, ND6^1^, pCytb and tRNA^Thr2^ might have independently occurred in each species, because the degeneration degrees and patterns were different among these species (Additional file 4). Alternatively, the degenerations could have originated before the divergence of the five species and then continued to accumulate numerous substitutions and deletions in each species. The deletion of tRNA^Glu1^ in the duplicate CR gene order could not have originated before the divergence of the *A. ibis*, *A. modesta* and *A. intermedia*, but likely occurred independently in two lineages, according to the evolutionary relationships among the five *Ardea* species shown in Figure 2, in which one is leading to *A. ibis* and the other is leading to *A. modesta* and *A. intermedia*,.

### Maintenance of the duplicated regions

According to the TDRL model, one copy of each duplicated gene or CR usually degenerates and is eventually lost during the evolutionary process [18]. However, persistent and stable duplications in mtDNA had been widely reported for several groups of vertebrates, such as fish [48, 49], frogs [20, 50], snakes [19, 51, 52], and birds [5, 7–15], and are usually interpreted to be concerted evolution. When duplications evolve in concert, paralogous sequences within individuals are more similar than orthologous sequences across closely related species [5, 47]. Likewise, in the ardeid mtDNAs with duplicate tRNA^Thr^–CR gene order, we could observed that all duplications including the partial Cytb, tRNA^Thr^, tRNA^Pro^, ND6, tRNA^Glu^ and CRs were nearly identical to each other within each individual, but very different between species (Additional file 3), suggesting that they were evolving in concert. In the mtDNAs of *Ardea* and *I. cinnamomeus*, the concerted evolution might have been stopped in some parts by large deletions, but still occurred between the duplicated CR.

The primary proposed mechanisms of concerted evolution include tandem replication slippage and gene conversion via general recombination [5, 19]. The tandem replication slippage sequentially homogenized sequences between duplications [19], therefore this mechanism cannot account for the observation in most ardeid species, where the homogenized duplications were interrupted by a divergent section nesting in the middle of CR domain I. In contrast, such uneven homogenized pattern could be easily created and maintained by gene conversion, similar to the previous reports in albatrosses [7, 12], the black-faced spoonbill [11], the ruff [13], boobies [14] and Philippine hornbills [15]. In albatrosses, Abbott et al. [7] proposed that the gene conversion could have multiple recombination points, whereby certain duplicated portions were regularly homogenized while intervening sections remained unaffected and therefore evolved independently. In boobies and Philippine hornbills, Morris-Pocock et al. [14] and Sammler et al. [15] seperately suggested that the concerted evolution in those birds was well consistent with a novel gene conversion model outlined by Kumazawa et al. [20] if the intervening divergent section corresponds to putative Replication Fork Barrier (RFB) and the replication in avian mtDNA is initiated from multiple points. Based on this model, the gene conversion is mediated by RFB regions in CRs, and the duplicated CRs are easier to recombine than other duplicated portions, which resulting more stable of the homogenized CRs. This explanation matched with the observation in the ardeid species, where the duplicated CRs were maintained in all species while extra copies of different genes were degenerating or deleted in some species. The conflict with this model in the ardeid birds was no observation of the divergence between putative RFBs in both *I. flavicollis* and *B. stellaris*. To explain such conflict observed in an individual of the Luzon Tarictic Hornbill (*Penelopides manillae*), Sammler et al. [53] postulated a possible scenario that RFB itself might have been subjected to homogenization via strand exchange once the replication fork is erroneously not halted at the RFB in the evolutionary history.

## Conclusions

In this study, the complete mtDNA of 16 ardeid birds were sequenced and four gene order types were found, including two that were newly discovered. These genomes strongly support the TDRL model of mt gene rearrangements and concerted evolution between duplications. Furthermore, based on the analysis of the phylogenetic relationships among the surveyed ardeid species using the concatenated nucleotide sequences of two rRNA and 12 protein coding genes, we found that our results were broadly congruent with previously studies using the DNA hybridization [44], Cytb [45] and vocalization [40]. We also found that most of the nodes in phylogenetic study were supported by high PP and BP values, suggesting that complete mt genomics could be promising molecular markers for resolving deep phylogenetic relationships in ardeid birds.

### Availability of supporting data

All supporting data is included as additional files. Complete mitochondrial DNA sequences have been deposited in NCBI GenBank under accession numbers KJ190944 to KJ190959.

## Electronic supplementary material

Additional file 1: **Primer sequences used in this study.** (DOCX 16 KB)

Additional file 2: **Schematic illustration of the strategy to amplify the region between Cytb and 12S rRNA.** Lines indicate PCR products. Corresponding primer pairs are shown under the lines. (TIFF 22 KB)

Additional file 3: **Alignments of the duplicated CR within individuals of sixteen ardeid birds.** *Dots* indicate identity of nucleotides to the reference sequence and *dashes* indicate gaps. The sequences of C stretch, TAS, F, E, D, C, BSB, B and CSB1 are highlighted in blue and are in bold. The sequences with long repeated motifs are highlighted in green and red. Eeul, *E. eulophotes*; Egar, *E. garzetta*; Esac, *E. sacra*; Abac, *A. bacchus*; Bstr, *B. striatus*; Nnyc, *N. nycticorax*; Bste, *B. stellaris*; Ifla, *I. flavicollis*; Ieur, *I. eurhythmus*; Isin, *I. sinensis*; Icin, *I. cinnamomeus*; Acin, *A. cinerea*; Apur, *A. purpurea*; Amod, *A. modesta*; Aint, *A. intermedia*; Aibi, *A. ibi*s. (PDF 118 KB)

Additional file 4: **Pair-wise alignments of subsections between the sizable no coding sections and corresponding functional genes within individuals of five species.** NC1: no coding section between tRNA^Thr^ and tRNA^Glu1^ or CR^1^; NC2: no coding section between CR^1^ and tRNA^Pro^. Numerals in brackets indicate the positions of the aligned sequences. Vertical lines indicate boundary of the gene. Dots indicate identity of nucleotides to the reference sequence; dashes indicate gaps. (PDF 31 KB)
