# Supplementary material for: The complete mitochondrial genomes of sixteen ardeid birds revealing the evolutionary process of the gene rearrangements
Source: BMC Genomics. 2014 Jul 8;15(1):573. doi: 10.1186/1471-2164-15-573 (PMC4111848; doi:10.1186/1471-2164-15-573)
Supplement: Supplementary file 1 — Additional file 1: Primer sequences used in this study. (DOCX 16 KB) [file 12864_2014_6274_MOESM1_ESM.docx]

| **No.** | **Primer name** | **Nucleotide sequences (5’–3’)** | **Reference** |
| --- | --- | --- | --- |
| **1** | **LACOIF** | **TCYATYGGCTCACTCATCTCAATAACAGCAG** | **This study** |
|  | **LA16SR** | **TGCACCATTAGGTTGTCCTGATCCAACATCGAGGT** | **[24]** |
| **2** | **LA12SF** | **CAAACTGGGATTAGATACCCYACTATGC** | **This study** |
|  | **LACOIIIR** | **GTCTACTATGTGGTARGARTGTGCTTGGTG** | **This study** |
| **3** | **Phe2F** | **AAAGCATGGCACTGAAGATGC** | **[22]** |
|  | **16S1R** | **TTTCATCTTTCCCTTACGGTAC** | **[22]** |
| **4** | **AR12SF** | **GGTAAGTCGTAACAAGGTAAGTG** | **This study** |
|  | **AR16SR** | **GACAATTGATTGCGCTAC** | **This study** |
| **5** | **AR16SF** | **TGATGCCTGCCCAGTGAC** | **This study** |
|  | **ARND1R** | **TGTGCTACTGCTCGTAGTG** | **This study** |
| **6** | **ARND1F** | **TAGCCATRTCTAGCCTRGCAG** | **This study** |
|  | **ND21R** | **GATGTCTCATTGTCCTGTGAATC** | **[22]** |
| **7** | **ARND2F** | **AACCAYTGARTCATAGCCTGAAC** | **This study** |
|  | **ARTyrR** | **GCGTTAGGCTGTAGTCCTTTTTAC** | **This study** |
| **8** | **ARCysF** | **ATGAGCTTGCAACTCAACATG** | **This study** |
|  | **ARCOIR** | **ATGTCTARTGAGGAGTTTGC** | **This study** |
| **9** | **ARCOIF** | **AACYGGCATYAAAGTCTTYAGCTG** | **This study** |
|  | **ARLysR** | **GGCTAGTGCTGTTRCATAGCTTC** | **This study** |
| **10** | **ARCOⅡF** | **ATYTTCTAYGGCCAATGCTCAG** | **This study** |
|  | **ARCOIIIR** | **CCTTGGAAYGTGCTYTCTCG** | **This study** |
| **11** | **ARCOIIIF** | **TACCAYATAGTAGACCCAAGC** | **This study** |
|  | **ARND4LR** | **GTTCGGGTGGAGGCTAC** | **This study** |
| **12** | **ARArgF** | **CAAACTCTATGACTTTCTCCATGTC** | **This study** |
|  | **ND41R** | **GATCAGTTGAATAGTGCGATTATG** | **[22]** |
| **13** | **ARND4F** | **AACACAAAYTAYGAACGCAC** | **This study** |
|  | **ND51R** | **CGAATTGGGCTGATTTTCCTG** | **[22]** |
| **14** | **ARND5F1** | **CCAYAAAYACCTGAGARATCCAAC** | **This study** |
|  | **ARCytbR** | **GAYCCRAAGTTYCATCAGGC** | **This study** |
| **15** | **ARND5F2** | **ATTGCCTCCCACCTAATCG** | **This study** |
|  | **ARThrR** | **CTTCATTCTTTGGYTTACAAGRCC** | **This study** |
| **16** | **Cytb3F** | **TGAGTAGGCAGCCAACCAGTAGA** | **[22]** |
|  | **ARCR742R** | **GCACAGTGTAAAAATGATTCC** | **This study** |
| **17** | **L537** | **CCTCTGGTTCCTCGGTCAG** | **[25]** |
|  | **ARProR** | **ATRCCAGCTTTGGGAGTTGG** | **This study** |
| **18** | **ARCR742F** | **GGAATCATYTTTACCCTGTGCAC** | **This study** |
|  | **CD1R** | **CTGACCGAGGAACCAGAGG** | **[22]** |
| **19** | **ARCR372F** | **CTCACGTGAAATCAGCAACC** | **This study** |
|  | **AR12SR1** | **AGGTTAGGACTAAGTCTTTTGTC** | **This study** |
