# Supplementary figures and images for: The complete mitochondrial genomes of sixteen ardeid birds revealing the evolutionary process of the gene rearrangements
Source: BMC Genomics. 2014 Jul 8;15(1):573. doi: 10.1186/1471-2164-15-573 (PMC4111848; doi:10.1186/1471-2164-15-573)

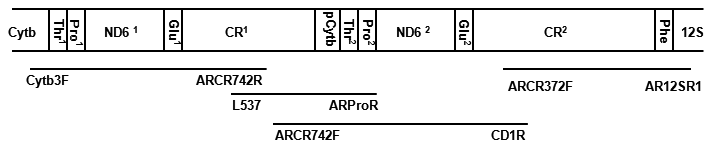

Supplement: Supplementary file 2 — Additional file 2: Schematic illustration of the strategy to amplify the region between Cytb and 12S rRNA. Lines indicate PCR products. Corresponding primer pairs are shown under the lines. (TIFF 22 KB) [file 12864_2014_6274_MOESM2_ESM.tiff]
