# Supplementary material for: The complete mitochondrial genomes of sixteen ardeid birds revealing the evolutionary process of the gene rearrangements
Source: BMC Genomics. 2014 Jul 8;15(1):573. doi: 10.1186/1471-2164-15-573 (PMC4111848; doi:10.1186/1471-2164-15-573)
Supplement: Supplementary file 3 — Additional file 3: Alignments of the duplicated CR within individuals of sixteen ardeid birds. Dots indicate identity of nucleotides to the reference sequence and dashes indicate gaps. The sequences of C stretch, TAS, F, E, D, C, BSB, B and CSB1 are highlighted in blue and are in bold. The sequences with long repeated motifs are highlighted in green and red. Eeul, E. eulophotes; Egar, E. garzetta; Esac, E. sacra; Abac, A. bacchus; Bstr, B. striatus; Nnyc, N. nycticorax; Bste, B. stellaris; Ifla, I. flavicollis; Ieur, I. eurhythmus; Isin, I. sinensis; Icin, I. cinnamomeus; Acin, A. cinerea; Apur, A. purpurea; Amod, A. modesta; Aint, A. intermedia; Aibi, A. ibis. (PDF 118 KB) [file 12864_2014_6274_MOESM3_ESM.pdf]

```
             C stretch            TAS                         TAS                    TAS
EeulCR1 CCCCCCCCCT TCCCCCCCCA TACATCTCAA CTGCTTCAGC AAGCTCTATG TATGGGCATG CATTGGTCTA TATG------ CCTCATTTTA CTTAACGATG  [ 100]
EeulCR2 .......... .......... .......... .......... .......... .......... .......T.G ..CAATTACA T..T...C.. TAC.T.A.CA  [ 100]
                TAS                   TAS          TAS
EeulCR1 CTAGGTATAC ATTTATATGC ------ATGT ACTAAGTCCA TGTACTGTAC TG-GTGGCAG AACTT--TAA TTCTCCACTG TT---CTACT CAGAGGATTA  [ 200]
EeulCR2 TC.A.C.C.T CCA...GCT. TTCCCT.... ....G.G... .AAGA..CTT GATA.AAT.T T.T..CT... .CG....T.A .AGAA...T. ..........  [ 200]
                                                TAS
EeulCR1 ATTCTGTAAT GAATTTAGGA ACAGTAACGT AACCTGTACT AAAACCATAG TTGAGGTGGG TTGTACATAT ACTCTGGAGT TAGGATACGG CAGTGCTTAA  [ 300]
EeulCR2 .......... .......... .......... .......... .......... .......... .......... .......... .......... ..........  [ 300]
                                                                                                         F box
EeulCR1 GCACTGGTAG GTGAATGGTA ACTGGCCATA GCAGTGCGAC TATCTCCTGA TGTGCCGGTA TCTGAAGTAC CAGGTTATTT ATTAGTCGGT CTTCTCACGT  [ 400]
EeulCR2 .......... .......... .......... ..G....... .......... .......... .......... .......... .......... ..........  [ 400]
                                                     E box                                                D box
EeulCR1 GAAATCAGCA ACCCGCCGCA TATAAGGCTC TACGTTACTA GCTTCAGGAC CATTCATTCC CCCTACACCC TAGCCCAACT TGCGCTTTTG CGCCTCTGGT  [ 500]
EeulCR2 .......... .......... .......... .......... .......... .......... .......... .......... .......... ..........  [ 500]
                                                              C box
EeulCR1 TCCTCGGTCA GGGCCATGCC TCGGTTTACT TAGCACTCGG TCCTCTTCAC AGAGTCATTT GGTTGATGCT TGTCTGCTTC TCACCCGTGA TCGCGGCATC  [ 600]
EeulCR2 .......... .......... .......... .......... .......... .......... .......... .......... .......... ..........  [ 600]

EeulCR1 TGGATTGCCT GGGGCGCCTC TAGTAATTTT CTTCTTCTAA ACTTCTTCAG GCTGCCCTCC GGTGCACCGC GGCGCAGCCA TCGAAGACGT GAGCATACAG  [ 700]
EeulCR2 .......... .......... .......... .......... .......... .......... .......... .......... .......... ..........  [ 700]
                                                                                           BSB           B box
EeulCR1 ACGCGTCATC GGCCTATTAT TCGTGGTCAG GCGTCACTGG ATGAGACGGT TGGAGTATTT GTGGAATCAT TTTTACACTG TGCACTTTGT TTTCCATTTG  [ 800]
EeulCR2 .......... .......... .......... .......... .......... .......... .......... .......... .......... ..........  [ 800]
                                                       CSB1
EeulCR1 GTTGTTGGTG TGTCCACTAA CCCCTAACAT GGTGTTATTT GATGAATGCT TGTTGGACAT ATTTTTTTCC ATATTCTACT TATTTTACTT CCTCTAATTT  [ 900]
EeulCR2 .......... .......... .......... .......... .......... .......... .......... .......... .......... ..........  [ 900]

EeulCR1 TCTTTGAGTT CCAAAACAAA ACTAGGTAAC TTTCAACTAA AAATTTAGCG ATCATATCAA AAAATTTTTA CGAATCATAT TCGTATATCT TACATTACCT  [1000]
EeulCR2 .......... .......... .......... .......... .......... .......... .......... .......... .......... ..........  [1000]
                                     7 x 81 bp + 1 incomplete repeat (57 bp) in CR1
EeulCR1 TAAAGCACTA AAATTTCATT AAAAATATAC TATACCCTTC ATGAACTGTA TCCTTATATC TTTACGTTAC CTTTACTGCT TTAAAGCACT AAAATTTCAT  [1100]
EeulCR2 .......... .......... .......... .......... .......... .......... C......... .......... .......... ..........  [1100]
                                     4 x 81 bp + 1 incomplete repeat (57 bp) in CR2
EeulCR1 TAAAAATATA CTATACCCTC CATGAACTGT ATCCTTATAT CTTTACGTTA CCTTTACTGC TTTAAAGCAC TAAAATTTCA TTAAAAATAT ACTATACCCT  [1200]
EeulCR2 .......... .........T .......... .......... .......... .......... .......... .......... .......... ..........  [1200]

EeulCR1 TCATGAACTG TATCCTTATA TCTTTACGTT ACCTTTACTG CTTTAAAGCA CTAAAATTTC ATTAAAAATA TACTATACCC TTCATGAACT GTATCCTTAT  [1300]
EeulCR2 .......... .......... .......... .......... .......... .......... .......... .......... .......... ..........  [1300]

EeulCR1 ATCTTTACGT TACCTTTACT GCTTTAAAGC ACTAAAATTT CATTAAGAAT ATACTATACC CTTCATGAAC TGTATCCTTA TATCTTTACG TTACCTTTAC  [1400]
EeulCR2 .......... C......... ...------- ---------- ---------- ---------- ---------- ---------- ---------- ----------  [1400]

EeulCR1 TGCTTTAAAG CACTAAAATT TCATTAAAAA TATACTATAC CCTTCATGAA CTGTATCCTT ATATCTTTAC GTTACCTTTA CTGCTTTAAA GCACTAAAAT  [1500]
EeulCR2 ---------- ---------- ---------- ---------- ---------- ---------- ---------- ---------- ---------- ----------  [1500]

EeulCR1 TTCATTAAAA ATATACTATA CCCTTCATGA ACTGTATCCT TATATCTTTA CGTTACCTTT ACTGCTTAA AGCACTAAAA TTTCATTAAA AATATACTAT  [1600]
EeulCR2 ---------- ---------- ---------- ---------- ---------- ---------- ------... .......... .......... ..........  [1600]

EeulCR1 ACCCTTCATG AACTGTATCC CTATAGCCTT TACCTTCAAC CCCCAAAATC CCACCAAAAA TAAATTAAAC GTACAAACTT CCACTACCCA CCACCCCATA  [1700]
EeulCR2 .......... .G........ .......... .......... .......... .......... .......... .......... .......... ..........  [1700]

EeulCR1 CAAAAATTAA ACGCCACGCT CAATGCAA-- ---------- ---------- ---------- ---------- ---------- ---------- ----------  [1800]
EeulCR2 .......... .......... ........TC AACCCTACAA CCCCCATCAA CCAATATCTA AACCCTACAC AATAAACAAC AAACAACAAA CAACAAACAA  [1800]

EeulCR1 ---------- ---------- ---------- ---------- ---------- ---------- ---------- ---------- ---------- ----------  [1900]
EeulCR2 TAAACAACAA ACAATAAACA ACAAACAATA AACAACAAAC AATAAACAAT AAACAATAAA CAACAAACAA TAAACAATAA ACTAAACAAT AAACAATAAA  [1900]

EeulCR1 ---------- ---------- ---------- ---------- ---------- ---------- ---------- ---------- ---------- ----------  [2000]
EeulCR2 CAACAAACAA TAAACAACAA ACAATAAACA GCAAACAATA AACAATAAAC AATAAACAAC AAACAATAAA CTAAACAATA AACAACAAAC AATAAACAAC  [2000]

EeulCR1 ---------- ---------- ---------- ---------- ---------- ---------- ---------- ---------- ---------- ----------  [2100]
EeulCR2 AAACAATAAA CAATAAACTA AACAATAAAC AATAAACAAC AAACAATAAA CAACAAACAA TAAACTAAAC AATAAACAAC AAACAATAAA CTAAACACTA  [2100]

EeulCR1 ---------- ---------- ------- [2127]
EeulCR2 AACAACAAAC AATAAACAAC AAACAAC [2127]


              C stretch           TAS                         TAS                    TAS
EgarCR1 CCCCCCCCTT CCCCCCCCAT ACATTTCAAC TGCTCAAACA AGCTCTATGT ATGGGCATGC ATTGGTCTAT ATG------C CTCATTCTGT CTAGTAATGT  [ 100]
EgarCR2 .......... .......... .......... .......... .......... .......... .....CT.G. ..AGCCACAT ..T..C..AC AC.CC.G.A.  [ 100]
          TAS    TAS                                     TAS                   TAS
EgarCR1 TAGATATACA T--------- ---TTACGTG C--------- ATGTACTAAG TCC---ATGC ACT-GTACTG TAGG------ ----CAAGAT -----CCTAA  [ 200]
EgarCR2 C.AGC...TC .ACATGTCTT AAAC...A.A .CCTTCTCCG ...A..C.G. ...TCT...T ...A.G..CA ..A.ATGCTT GATAT..T.. TATTT.T...  [ 200]
```

```
                                                                                                       TAS
EgarCR1 TT-TCCACTG CT---CTACT TAGAGGACTA ATCCTGTAAT GAGCTTAGGA AAGGTAACGT AACCTGTACT AGAACCATGG TTAGGTGGGC TGTACATGTA  [ 300]
EgarCR2 .CG....T.A TAGAGT..T. .......... .......... .......... .......... .......... .......... .......... ..........  [ 300]

EgarCR1 CTTTGGAGTA AAGGTACGGC AGTGCTTGAG CAGTGGTAAG TGAATGGTAA CTGGCCATAG CAGTGCAACT ATCTCCTGAT GTGCCGGTAT CTGAAGTACC  [ 400]
EgarCR2 .......... .......... .......... .......... .......... .......... .......... .......... .......... ..........  [ 400]
                                 F box                                    E box
EgarCR1 AGGTTATTTA TTAGTCGGTC TTCTCACGTG AAATCAGCAA CCCGCCGCAT ATAAGGCTCT ACGTTACTAG CTTCAGGACC ATTCATTCCC CCTACACCCT  [ 500]
EgarCR2 .......... .......... .C........ .......... .......... .......... .......... .......... .......... ..........  [ 500]
                                  D box                                             C box
EgarCR1 AGCCCAACTT GCGCTTTTGC GCCTCTGGTT CCTCGGTCAG GGCCATGGCT CGGTTTACTT AGCACTCGGT CCTCTTCACA GAGTCATTTG GTTGATGCTT  [ 600]
EgarCR2 .......... .......... .......... .......... .......... .......... .......... .......... .......... ..........  [ 600]

EgarCR1 GTCTGCTTCT CACCCGTGAT CGCGGCATCT GGATTGCCTG GGGCGCCTCT AGTAATTTTC TTCTTCTAAA CTTCTTCAGG CTGCCCTCCG GTGCACCGCG  [ 700]
EgarCR2 .......... .......... .......... .......... .......... .......... .......... .......... .......... ..........  [ 700]

EgarCR1 GCGCTGCCAT CGAAGACGTG AGCATACAGA CGCGTCATCG GCCTATTATT CGTGGTCAGG CGTCACTGGA TGAGACGGTT GGAGTATTTG TGGAATCATT  [ 800]
EgarCR2 .......... .......... .A........ .......... .......... .......... .......... .......... .......... ..........  [ 800]
             BSB                 B box                                  CSB1
EgarCR1 TTTACACTGT GCACTTTGTT TTCCATTTGG TTGTTGGTGT GTCCACTAAC CCCTAACATG GTGTTATTTG ATGAATGCTT GTTAGACATA TTTTTTTCCA  [ 900]
EgarCR2 .......... .......... .......... .......... .......... .......... .......... .......... .......... ..........  [ 900]

EgarCR1 CTTTCTACTT ATTTTACTTC CTCTAATTTT CTTTGAGTTC CAAAACACAA CTAGGTAACT TTCAACTAAA AATTTAACGA GTATATCAAA AAATTTTTAC  [1000]
EgarCR2 .......... .......... .......... .......... .......... .......... .......... .......... .......... ..........  [1000]
                                                     5 x 81 bp + 1 incomplete repeat (77 bp) in CR1
EgarCR1 AAATCATATT CGTATACCTT ACATTACTTT AAAGCACTAA AATTTCATTA AAAATATACT ATACCCTTCA TGAACTGTAT CCTTATATCT TTATGTTACC  [1100]
EgarCR2 .......... .......... .......... .......... .......... .......... .......... .......... .......... ..........  [1100]
                                                     3 x 81 bp + 1 incomplete repeat (77 bp) in CR2
EgarCR1 TTTACTATTT TAAAGCACTA AAATTTCATT AAAAATATAC TATACCCTTC ATGAACTGTA TCCTTATATC TTTATGTTAC CTTTACTACT TTAAAGCACT  [1200]
EgarCR2 ......C... .......... .......... .......... .......... .......... .......... .......... .......... ..........  [1200]

EgarCR1 AAAATTTCAT TAAAAATATA CTATACCCTT CATGAACTGT ATCCTTATAT CTTTATGTTA CCTTTACTAC TTTAAAGCAC TAAAATTTCA TTAAAAATAT  [1300]
EgarCR2 .......... .......... .......... .......... .......... .......... .......--- ---------- ---------- ----------  [1300]

EgarCR1 ACTATACCCT CCATGAACTG TATCCTTATA TCTTTATGTT ACCTTTACTA CTTTAAAGCA CTAAAATTTC ATTAAAAATA TACTATACCC TTCATGAACT  [1400]
EgarCR2 ---------- ---------- ---------- ---------- ---------- ---------- ---------- ---------- ---------- ----------  [1400]

EgarCR1 GTATCCTTAT ATCTTTATGT TACCTTTACT ACTTTAAAGC ACTAAAATTT CATTAAAAAT ATACTATACC CTTCATGAAC TGTATCCTTA TATCTTTATG  [1500]
EgarCR2 ---------- ---------- ---------. .......... .......... .......... .......... .......... .......... ..........  [1500]

EgarCR1 TTACCTCAAC CCCCAAAATC CAATCAAAAA TAAATTAAAC GAACAAACTT CACCACCCAC CACCCCATAC AAAAATTAAA CACCACGCTC AATGCAAC--  [1600]
EgarCR2 .......... .......... .......... .......... .......... .......... .......... .......... .......... ........CA  [1600]

EgarCR1 ---------- ---------- ---------- ---------- ---------- ---------- ---------- ---------- ---------- ----------  [1700]
EgarCR2 ACCCCACAGC CCCCATCAAC CAACATCAAA CCCTGCACAA CAAACAATAA ACAACAAACA ATAAACAACA AACAACAAAC AACAAACAAC AAACAACAAA  [1700]

EgarCR1 ---------- ---------- ---------- ---------- ---------- ---------- ---------- ---------- ---------- ----------  [1800]
EgarCR2 CAACAAACAA CAAACAATAA ACAACAAACA ATAAACAACA AACAACAAAC AATAAACAAC AAACAACAAA CAATAAACAA CAAACAATAA ACAACAAACA  [1800]

EgarCR1 ---------- ---------- ---------- ---------- ---------- ---------- ---------- ---------- ---------- ----------  [1900]
EgarCR2 ATAAGCAACA AACAACAAAC AACAAACAAC AAACAATAGA CAACAAACAA CAAACAACAA ACAACAAACA ATAAACAACA AACAACAAAC AACAAACAAC  [1900]

EgarCR1 ---------- ---------- ---------- ---------- ---------- ---------- ---------- ------- [1977]
EgarCR2 AAACGACAAA CAATAAACAA CAAACAACAA ACAATAAACA ACAAACAACA AACAATAAAC AACAAACAAC AAACAAC [1977]


           C stretch                                          TAS                        TAS
EsacCR1 CCCCCCCCCT TCCCCCCCCA TACATTTCAA CTGCTTCAAC AAGCTCTATG TATGGGCATG CATTGGTCTA TATG------ CCTCATCTTG CTCAGTAATG  [ 100]
EsacCR2 -......... .......... .......... .......... .......... ....A.T... ....AACT.G ..CAATTACA T.....TC.A TA..CC..CA  [ 100]
      TAS            TAS                                         TAS                TAS
EsacCR1 TTAGATATAC ATTTAT---- ------ATG- ---------- ---------- ----TATGTA CTAAGTCCAT ACGATGT--- ACTGAGGACA GAATCTTAAT  [ 200]
EsacCR2 .C.A.C...T C.G...GCCT TAAACT...C ACTCTTCTCC ATGCACCAGA TCCC...... ...G.G.... .A....CTTG .TAT.AT.TT ATT.......  [ 200]
                                                                                                       TAS
EsacCR1 TCTCCACTGT T---TTACTT AGAGGATTAA TTCTGTAATG GATCCAGGAA TAGCAGCGTA ACCTGTACTA AAACCATGAT TAAGGTGGGT TGTACATACG  [ 300]
EsacCR2 CG....T.A. AAGA.....C .......... .......... .......... .......... .......... .......... .......... ..........  [ 300]

EsacCR1 CTCTGGAGCG AAGATACGGC AGTGCTTGAG CAGTGATAAG TGAATGGTAA CTGGCCATAG CAGTGCAGCT GTCTCCTGAT GTGCCGGTAT CTGAAGTACC  [ 400]
EsacCR2 .......... .......... .......... .......... .......... .......... .......... .......... .......... ..........  [ 400]
                                 F box                                   E box
EsacCR1 AGGTTATTTA TTGGTCGGTC TTCTCACGTG AAATCAGCAA CCCGCCGCAT ATAAGGCTCT ACGTTACTAG CTTCAGGACC ATTCATTCCC CCTACACCCT  [ 500]
EsacCR2 .......... .......... .......... .......... .......... .......... .......... .......... .......... ..........  [ 500]
                                  D box                                             C box
EsacCR1 AGCCCAACTT GCGCTTTTGC GCCTCTGGTT CCTCGGTCAG GGCCATGTCT CGGTTTACTT AGCACTCGGT CCTCTTCACA GAGTCATTTG GTTGATGCTT  [ 600]
EsacCR2 .......... .......... .......... .......... .......... .......... .......... ........T. .......... ..........  [ 600]
```

```
EsacCR1 GTCTGCTTCT CACCCGTGAT CGCGGCATCT GGATTGCCTG GGGCGCCTCT AGTAATTTTC TTCTTCTAAA CTTCTTCAGG CTGCCCTCCG GTGCACCGCG  [ 700]
EsacCR2 .......... .......... .......... .......... .......... .......... .......... .......... .......... ..........  [ 700]

EsacCR1 GCGCTGCCAT CGAAGACGTG AGCATACAGA CGCGTCATCG GTCTCTTATT CGTGGTCAGG CGTCACTGGA TGAGACGGTT GAAGTATTTG TGGAATCATT  [ 800]
EsacCR2 .......... .......... .......... .......... .......... .......... .......... .......... .......... ..........  [ 800]
           BSB                   B box                                        CSB1
EsacCR1 TTTACACTGT GCACTTTGTT TTCCATTTGG TTGTTGGTGT GTCCACTAAC CCCTAACATG GTGTTATTTG ATGAATGCTT GTTGGACATA TTTTTTTCCA  [ 900]
EsacCR2 .......... .......... .......... .......... .......... .......... .......... .......... .......... ..........  [ 900]

EsacCR1 TATTCTACTT ATTTTACTTC CTCTAATTTT CTTTGAGTTC TAAAACAAAA CTAGGTAACT TTCAACTAAA AATTTAGCGA GTATATCAAA AAATTTTTAC  [1000]
EsacCR2 ......G... .......... .......... .......... .......... .......... .......... .......... .......... ..........  [1000]
                                                                 3 x 81 bp + 1 incomplete repeat (69 bp)
EsacCR1 GAATCATATT CGTATATCTT ACATTACCTT AAAGCACTAG AATTTCATTA AAAATATACT ATACCCTTCA TGAACTGTAT CCTTATATCT TTACGTTACC  [1100]
EsacCR2 .......... .......... .......... .........A .......... .......... .......... .......C.. ......G... ..........  [1100]

EsacCR1 TTTACTGCTT TAAAGCACTA AAATTTCATT AAAAATATAC TATACCCTTC ATGAACTGTA TCCTTATATC TTTACGTTAC CTTTACTGCT TTAAAGCACT  [1200]
EsacCR2 .......... .......... .......... .......... .......... .......... .......... .......... .......... ..........  [1200]

EsacCR1 AAAATTTCAT TAAAAATATA CTATACCCTT CATGAACTGT ATCCTTATAT CTTTACGTTA CCTTTACTGC TTTAAAGCAC TAAAATTTCA TTAAAAATAT  [1300]
EsacCR2 .......... .......... .......... .......... .......... .......... .......... .......... .......... ..........  [1300]

#EsacCR1 ACTATACCCT TCATGAACTG TATCCTTATA CCTTTACCTT CAACCCCCAA AACCCCATCA AAAATAAATT AAACGTACAA GCTTCCACCC CCACCATCCC  [1400]
#EsacCR2 .........C ........C. .......... .......... .......... .......... .......... ........G. .......... ..........  [1400]

EsacCR1 ATGCAAAATT AAACGCCACG CTCAATGCAA ---------- ---------- ---------- ---------- ---------- ---------- ----------  [1500]
EsacCR2 .......... .......... .......... TCAACCCTAC AGGCCCCATC AACCAATATC TAACCCCACA CAATAAACAA CAAACAACAA ACAATAAACA  [1500]

EsacCR1 ---------- ---------- ---------- ---------- ---------- ---------- ---------- ---------- ---------- ----------  [1600]
EsacCR2 ATAAACAATA AACAACAAAC AATCAAACAA TAAACAATAA ACAATAAACA ATAAACAATA AACAATAAAC AATAAACAAC AAACAACAAA CAACAAACAA  [1600]

EsacCR1 ---------- ---------- ---------- ---------- ---------- ---------- ---------- ---------- ---------- ----------  [1700]
EsacCR2 CAAACAATAA ACAATAAACA ATAAACAATA AACAATAAAC AATAAACAAT AAACAATAAA CAATAAACAA TAAACAATAA ACAATAAACA ATAAACAATA  [1700]

EsacCR1 ---------- ---------- ---------- ---------- ---------- ---------- ---------- ---------- ---------- ----------  [1800]
EsacCR2 AACAATAAAC AATAAACAAT AAACAACGTC CTTGTAGCTT AACACCAAAG CATAGCACTG AAGATGCTAA GACGGATGCT CACATTCCCA AGGACAAAAG  [1800]

EsacCR1 ----------  [1810]
EsacCR2 ACTTAGTCCT  [1810]


                                                                       C stretch
AbacCR1 CCTACTCCAC CCCACACCAA AAACACCTTA TAATGTACTC TCCTACATTA TGTACCCCCC CTTACCCCCC CACACAATAA CTCTGCTCTA CAAGCAAGTT  [ 100]
AbacCR2 -......... .......... .......... .......... .......... .......... .......... .......... .......... ..........  [ 100]
           TAS                    TAS                                    TAS          TAS                       TAS
AbacCR1 GTATGTATGG CTATGCATTA ACTTATATCC CTCATTAGCA GGTAATGTTA AGTTATACAT TCAAGTGTAT GTACTAGGTC CATAGGATGC CTGATGTAAT  [ 200]
AbacCR2 .......... TC........ .........T .C.....A.. ..C....... .......... .T........ .....GT... ...-.AC.AT G.TC.T.CG.  [ 200]

AbacCR1 GTCATTTCTT AGTCGTTCAA TAAGCTTTCA GAGGAT-TAA TCT--TGTAA TTAATTTAGG AATGGCTTCA TTATTCTGTA CTAAACCCAT TAGCTGATGA  [ 300]
AbacCR2 ACA.G..... G..T.....T .TTCTA...T TG.T.CG... AT.AA....C ...G.C.GT. G..A.T...G A...AT.... .......... ..........  [ 300]

AbacCR1 GGTGTACATA TTAAGTGGTT TCTAGTAAAT GACTGTGCTT GGGTTAAGTA AGTCAATGGT AGCAGACATG GTAATTCAAT CATCTCTTGA TGTGCCGGTC  [ 400]
AbacCR2 .......... .......... .......... .......... .......... .......... .......... .......... .......... ..........  [ 400]
                                          F box                                            E box
AbacCR1 TCTAAAGTGT TAGGTTATTT ATTAATCGTC CTTCTCACGT GAAATCAGCA ACCCGGTGTA GATAAGGTTT TACGTTACTA GCTTCAGGAC CATTCATTCC  [ 500]
AbacCR2 .......... .G........ .......... .......... .......... .......... .......... .......... .......... ..........  [ 500]
                                             D box                                                C box
AbacCR1 CCCTACACCC TAGCACAACT TGCTCTTTTG TACCTCTGGT TCCTCGGTCA GGGCCATATC TCGGTTGACT TAGCACTCGG TCCTCTTCAC AGAGTCATTT  [ 600]
AbacCR2 .......... .......... .......... .......... .......... .......... .......... .......... .......... ..........  [ 600]

AbacCR1 GGTTGATGCT TGTCTGCTTC TCACCCGTGA TCGCGACATC TGGATTGCCT GGGGCACCTC TAGTAATTTT TTTCTTCTAA ACTTCTTCAG GCAGCCCTCC  [ 700]
AbacCR2 .......... .......... .......... .......... .......... .......... .......... .......... .......... ..........  [ 700]

AbacCR1 GGTGCACCGC GGCGCAGCCA TCGAAGACTG TGAGCATTTA GGACGCGTCA TTGGACTCTT ATTAGCTCTC AGGAATGACT GGATGAGACG GTTTGAGTGT  [ 800]
AbacCR2 .......... .......... .......... .......... .......... .......... .......... .......... .......... ..........  [ 800]
                             BSB               B box                                                  CSB1
AbacCR1 TTGTGGAATC ATTTTTACAC TGTGCACTTG CTATTTCATT TGGTTGTTGG TGTGTCCACT ATCCCCAAAT ATGGTGTTAT TTGGTGAATG CTTGTTGGAC  [ 900]
AbacCR2 .......... .......... .......... .......... .......... .......... .......... .......... .......... ..........  [ 900]

AbacCR1 ATAATTTTAC CTACACTTTA CCTATTTACA CTTCCTCTAA TTTCCTTTGA CTTCTTAAAC AAGACTAGGT AAATTTCAAC TAAAAATTTA ACAAGTCTTA  [1000]
AbacCR2 .......... .......... .......... .......C.. .......... .......... .......... .......... .......... ..........  [1000]

AbacCR1 TCAAAAATTT TTTCACATCT TATTCTTATA TTTTACATTT CCTTCACCCA CTGGAGTTAC ATTAAAAAAA CATACCATAA ACAACGTAAA TTTTTCGACG  [1100]
AbacCR2 .......... .......... .......... .......... .......... .......... .......... .......... .......... ..........  [1100]
```

```
AbacCR1 TGTTATTATA TATTTGTGCA CAATTATCAC TATTCACACT GCTGAAGTTA CATTAAAAAA ATAAACATTA CTATGCTTAG CACAACTAA- ----------  [1200]
AbacCR2 .......... .......... .......... .......... .......... .......... ........C. .......... .........A CTCTTGCATC  [1200]

AbacCR1 ---------- ---------- ---------- ---------- ---------- ---------- ---------- ---------- ---------- ----------  [1300]
AbacCR2 GCTTCCCCAA ACTTATAACA AAACTAAACA AACAACCAAC TTAAACAACA AACAAGCAAC AAACAAGCAA CAAATAAGCA ACAAACAAGC AACAAACAAG  [1300]

AbacCR1 ---------- ---------- ---------- ---------- ---------- ---------- ---------- ---------- ---------- ----------  [1400]
AbacCR2 CAACAAACAA GCAACAAACA AGCAACAAAC AAGCAACAAA CAAGCAACAA ACAAGCAACA AACAAGCAAC AAACAAGCAA CAAATAAGCA ACAAATAAGC  [1400]

AbacCR1 ---------- ---------- ---------- ---------- ---------- ---------- ---------- ---------- ---------- ----------  [1500]
AbacCR2 AACAAATAAG CAACAAACAA GCAACAAATA AGCAACAAAC AAGCAACAAA CAAGCAACAA ATAAGCAACA AACAAGCAAC AAATAGGCAA CAAACAAGCA  [1500]

AbacCR1 ---------- ---------- ---------- ---------- ---------- ---------- ---------- ---------- ---------- ----------  [1600]
AbacCR2 ACAAATAAGC AACAAACAAG CAACAAACAA GCAACAAACA AGCAACAAAT AAGCAACAAA CAAGCAACAA ACAAGCAACA AACAAGCAAC AAACAAGCAA  [1600]

AbacCR1 ---------- ---------- ---------- ---------- ---------- ---------- ---------- ---------- ---------- -- [1692]
AbacCR2 CAAACAAGCA ACAAATAAGC AACAAATAAG CAACAAACAA GCAACAAATA AGCAACAAAT AAGCAACAAA TAAGCAACAA ATAACGAACA CC [1692]


                   C stretch      TAS                                    TAS                    TAS
BstrCR1 CTGCTACCCC CCCTTACCCC CCCATACATT CAAACTAGTT AGGTAGGGTC GTTATGTATG GGTACAGTTT CTATGTATAG GTGTACATTG GTTTACAGGC  [ 100]
BstrCR2 .......... .......... .......... T.......A. .A..C.---- ---------- ---------- ........GA .CA.G....A AC...T.T..  [ 100]
                                   TAS                                TAS
BstrCR1 CCCTATATAA GTACAATGCT GGGAGTACAT TAGGACGCAC GTATAACCTA TCGAGCATCA CGGAAAAGCT ATGCACGAAG TCCATTGGAT GCCTGATATA  [ 200]
BstrCR2 .....-G..G .C-T....T. AA-------- ---------- ---------. .TATA...T. TA.TG----C ...T..T..T ......TT.. .T..TTCG..  [ 200]

BstrCR1 -ATTTCTTTC CTTGACCGTT CTAAGCTTTC AGGGGATTAA TCTGTACTTA ACTTAGGAAT GATTCCATAA CCTGTACTAA AACCATAGTA ACAGTGGATT  [ 300]
BstrCR2 C.AGCTC..A A.C.T..A.. ..TCTA...T .......... .......... .......... .......... .......... .......... ..........  [ 300]

BstrCR1 GTATATGAGT AGTGGATTGA GTGTACGGCT GTGCTTGAAC ACAGTGATTG AATGGTAGCA GGCCATGCTA GCTCAATAAT CTCTTGAAGT ACCGGTATCT  [ 400]
BstrCR2 .......... .......C.. .......... .......... .......... .......... .......... .......... .......... ..........  [ 400]
                                         F box                                            E box
BstrCR1 GAAGTACCAG GTGATTTATT AATCGTTCCT CTCACGTGAA ACCAGCAACC CGTTGCATAA AATGCTCTAC ACGACTAGCT TCAGGATCAT CCTTTCCCCC  [ 500]
BstrCR2 .......... .......... .......... .......... .......... .......... .......... .......... .......... T.........  [ 500]
                                           D box                                               C box
BstrCR1 TACACCCTAG CCCAACTTGC GCTTTTGCGC CTCTGGTTCC TCGGTCAGGG CCATGGCTCG GTTTACTTAG CACTCGGTCC TCTTCACAGA GTTATTTGGT  [ 600]
BstrCR2 .......... ...G...... .......... .......... .......... .......... .......... .......... .......... ..C.......  [ 600]

BstrCR1 TGATGCTTGT CTGCTTCTCA CCCGTGATCG CGACATCTAG ATTGCCTGGG GCCCCTCTAG TATTTTTTCT CTTCTGGGTA ACTTCAGTGT GCCCCCCGGT  [ 700]
BstrCR2 .......... .......... .......... .......... .......... ..G....... .......... .......... .......... ..........  [ 700]

BstrCR1 GGATCGCGGC GCAGCCATCG AAGACTGTGG ACCCACAGAC GCGTCATCGG TCTCTTATTA GCTTTCAGGA ATGACTGGAT GAGACGGTTG GAGTATTTGT  [ 800]
BstrCR2 .......... .......... .......... .......... .......... ......G... .......... .......... .......... ..........  [ 800]
                      BSB                  B box                                         CSB1
BstrCR1 GGAATCATCT TTACCCTGTG CACTTTGTTT TCCATTTGGT TGTTGGCGTG TCCACTATCC CCAAACATGG TGCTATTTGA TGAATGCTTG TTGGACATAA  [ 900]
BstrCR2 .......... .......... .......... .......... .......... .......... .......... .......... .......... ..........  [ 900]

BstrCR1 TTCTACTTAC TTTCTTCTTA TTTACACTTC CTCTAATTTC CTTTCATTTG ATTAACAACA CTAGGCAAAT TTCAACTAAA AATTTAACAA GCCTTGTTGA  [1000]
BstrCR2 .......... .......... .......... .......... .......... ....G..... .......... .......... .......... ........A.  [1000]

BstrCR1 AAATTTTTCA CAAATCTTAT TCTTATATTT TACATTACTT TACACCACTG GAGTTACATT AAAAAACATG CCTTAAACAG CAAACTTTTT TTGGCGTGTT  [1100]
BstrCR2 .......... .......... .......... .......... .......... .G........ .......... .......... .......... ..A.......  [1100]

BstrCR1 ATTATATATT TACACATAAT TATTACCTTC CACACCACTG GAGTTACATT AAAAAAAATA AGCATTTTTA TGCTTAACAT GATCAAATTT TATGTT----  [1200]
BstrCR2 .......... .......... .......... .......... ...C.....C .......-.. .......... .......... .......... ......CCCT  [1200]

BstrCR1 ---------- ---------- ---------- ---------- ---------- ---------- ---------- ---------- ---------- ----------  [1300]
BstrCR2 CTATACTAAC CAGCACTAAA ATTTCAACCA AACAACAACG AACAACGAAC GACGAACAAC GAACGAACAA CAACGAACAA CGGACGAACA ACAACGAACA  [1300]

BstrCR1 ---------- ---------- ---------- ---------- ---------- ---------- ---------- ---------- ---------- ----------  [1400]
BstrCR2 ACGAACAAAC AACAACGAAC AACAAACAAC GAACAACGAA CGAACAACAA CGAACAACGA ACAAACAACA ACGAACAACA AACAACGAAC AACAACAAAC  [1400]

BstrCR1 ---------- ---------- ---------- ---------- ---------- ---------- ---------- -  [1471]
BstrCR2 AACGAACAAC AAACAACAAC AAACAACGAA CAACGAACAA CGAACAAACA ACAACGAACA ACGAACCCAC C  [1471]


                                              C stretch      TAS                        TAS
NnycCR1 ACTACCCCAA CAGTAAACAG TACGCTCAAT CTAGCTACCC CCCCTTACCC CCCCATACAT CAACTGCTTA GGCAGGGCTG TATGTATGGC CATGCATTGG  [ 100]
NnycCR2 .......... .......... .......... .......... .......... .......... .......... .......... .......... ..........  [ 100]
          TAS                 TAS              TAS                                        TAS
NnycCR1 TCTATATGCC CCATGCATTA TGTAATGTTA GAGTATACAT TTATATTTAT GTACGGTACC CACAGAATGT TTGATGTAAT ATTTTGTTCT TAGCTGTTCA  [ 200]
NnycCR2 .......... T....G...G ..C....... ..C.TC.T.G .----.CA.. ....T.AT.T ..T.AG..A. .CT-...T.C .A---.A.A. ..A.....T.  [ 200]
```

```
NnycCR1 TTCAT-TCTT TCAGGGGATT AATCCTGTAC TGTATTTAGG AATGGCTTCA TAATCTGTAC TAAAACCATG GTTCGTTGAG CTGTGCATAG TAAGTGGATC  [ 300]
NnycCR2 C.AGCG.A.C .T........ .......... .......... .......... .......... .......... .......... .......... ..........  [ 300]

NnycCR1 GAAGTGTACG GCTGTGCTTA AGCAAGTTAA CTGCAATGGT AGCTGGCCAT GGAAGTTCAA CTGTCACCTG ATGTGCCGGT ATCTGAAGTA CCAGGTTATT  [ 400]
NnycCR2 .......... .......... .......... .......... .......... .......... ..A..T.... .......... .......... ..........  [ 400]
                                F box                                       E box
NnycCR1 TATTGGTCGG TCTTCTCACG TGAAATCAGC AACCCGCCGC ATATAAGGCT CTACGTTACT AGCTTCAGGA CCATTCTTTC CCCCTACACC CCTAGCCCGA  [ 500]
NnycCR2 .......... .......... .......... .......... .......... .......... .......... .......... .......G.. ..........  [ 500]
                                  D box                                                C box
NnycCR1 CTTGCTCTTT TGCGCCTCTG GTTCCTCGGT CAGGGCCATA GCTCGGTTGA CTTAGCACTC AGTCCTCTTC ACAGAGTCAT TTGGTTGATG CTTGTCTGCT  [ 600]
NnycCR2 .......... .......... .......... .......... .......... .......... .......... .......... .......... ..........  [ 600]

NnycCR1 TCTCACCCGT GATCGCGACA TCTGGATTGC CTGGGGCGCC TCTAGTAATT TTTATCTTCT AAACTTCTTC AGGCAGCCCT CCGGTGCACC GCGGCGCAGC  [ 700]
NnycCR2 .......... .......... .......... .......... ...G...... .......... .......... .......... .......... ..........  [ 700]

NnycCR1 CATCGAAGAC TGTGAGCATA CAGACGCGTC ATCGGCCTAT TATTCGAGAG CGGGGAGCCA CTGGATGAGA CGGTTGGAGT ATTGGTGGTA TCATCTTTAC  [ 800]
NnycCR2 .......... .......... .......... .......... .......... .......... .......... .......... .......... ..........  [ 800]
           BSB                      B box                                      CSB1
NnycCR1 ACTGTGCACT TTGTTTTCCA TTTGGTTGTT GGTGTGTCCA CTAACCCCAA ACATGGTGCT ATTTGGTGAA TGCTTGTTGG ACATAATTTT ACCTTAACCT  [ 900]
NnycCR2 .......... .......... .......... .......... .......... .......... .......... .......... .......... ..........  [ 900]

NnycCR1 TACTAATTTA CACTTCCTCT AATTTCCTTT AACTTCACTT TGCAAATGTA GGAACTTTCA TCTAAAAATT TAACAAACTT TTTTAAAAAT TTTTTACAAA  [1000]
NnycCR2 .......... .......... .......... .......... .......... .......... .......... .......... .......... ..........  [1000]

NnycCR1 TTTTATTCTT ATCTTTTACA TTACCTTAAA CCACTGGAGT TACATTAAAA AATATACCAT AAACAACACA AACTTTTTCG ACGTGTTATT ATATATTTGT  [1100]
NnycCR2 .......... .......... .......... .......... .......... .......... .......... .....C.... .......... T.........  [1100]

NnycCR1 GCACATTTAT CACTCTTCTT ACCACTGGAG TTACATTAAA AAAAAACAAA CAATTATTAC ACTTAGTGTA ACCAAATTTT ATGTCTTAAC ACA-------  [1200]
NnycCR2 .......... .......... .......... .......... .......... .......... .......... .......... .......... ...GACACAC  [1200]

NnycCR1 ---------- ---------- ---------- ---------- ---------- ---------- ---------- ---------- ---------- ----------  [1300]
NnycCR2 TTCCACCAAA CCCCATTAAC TTTTCAAACA ACAAACCAAA CTATACATCT AAGCAGCGAA TACGAACGAA TAACGAACGA ATAACGAACG AATAACGAAC  [1300]

NnycCR1 ---------- ---------- ---------- ---------- ---------- ---------- ---------- ---------- ---------- ----------  [1400]
NnycCR2 GAATAACGAA CGAATAACGA ACGAATAACG AACGAATAAC GAACGAATAA CGAACGAATA ACGAACGAAT AACGAACAAA CAATGAACGA ATAACGAACA  [1400]

NnycCR1 ---------- ---------- ---------- ---------- ---------- ---------- ---------- ---------- ---------- ----------  [1500]
NnycCR2 AACAATGAAC GAATAACGAA CAAACAATGA ACGAATAACG AACGAATAAC GAACGAATAA CGAACGAATA ACGAACGAAT AACGAACAAA CAATGAACGA  [1500]

NnycCR1 ---------- ---------- ---------- ---------- ---------- ---------- ---------- ---------- ---------- ----------  [1600]
NnycCR2 ATAACGAACA AACAATGAAC GAATAACGAA CAAACAATGA ACGAATAACG AACAAACAAT GAACGAATAA CGAACAAACA ATGAACGAAT AACGAACGAA  [1600]

NnycCR1 ---------- ---------- ---------- ---------- ---------- ---------- ---------- ---------- ---------- ----------  [1700]
NnycCR2 CAATGAACGA ATAACGAACA AACAATGAAC GAATAACGAA CAAACAATGA ACGAATAACG AACAAACAAT GAACGAATAA CGAACAAACA ATGAACGAAT  [1700]

NnycCR1 ---------- ---------- ---------- ---------- ---------- ---------- ---------- ---------- ---------- ----------  [1800]
NnycCR2 AACGAACAAA CAATGAACGA ATAACGAACA AACAATGAAC GAATAACGAA CAAACCATGA ACGAATAACG AACAAACAAT GAACGAATAA CGAACAAACA  [1800]

NnycCR1 ---------- ---------- ---------- ---------- ---------- ---------- ---------- ---------- ---------- ----------  [1900]
NnycCR2 ATGAACGAAT AACGAACAAA CAATGAACGA ATAACGAACA AACAATGAAC GAATAACGAA CAAACAATGA GCGAATAACG AACAAACAAT GAACGAATAA  [1900]

NnycCR1 ---------- ---------- ---------- ---------- ---------- ---------- ---------- ---------- ---------- ----------  [2000]
NnycCR2 CGAACAAACA ATGAACGAAT AACGAACAAA CAATGAACGA ATAACGAACA AACAATGAAC GAATAACGAA CAAACAATGA ACGAATAACG AACAAACAAT  [2000]

NnycCR1 ---------- ---------- ---------- ---------- ---------- ---------- ---------- ---------- ---------- ----------  [2100]
NnycCR2 GAGCGAATAA CGAACAAACA ATGGACGAAT AACGAACAAA CAATGAACGA ATAACGAACA AACAATGAAC GAATAACGAA CAAACAATGA ACGAATAACG  [2100]

NnycCR1 ---------- ---------- ---------- ---------- ---------- ---------- ---------- ---------- ---------- ----------  [2200]
NnycCR2 AACAAACAAT GAACGAATAA CGAACAAACA ATGAACGAAT AACGAACAAA CAATGAGCGA ATAACGAACA AACAATGAAC GAATAACGAA CAAACAATGA  [2200]

NnycCR1 -------- [2208]
NnycCR2 ACGAATAA [2208]


                                                               C stretch                          TAS
BsteCR1 CCACTAAAAC CTTTAAAGCC ACCCTCACCC CCACAAATTT GCATTACCCC CCCTACCCCC CCAATAAACT TGGGTTATTA CATGTATGGG CATGCATTAA  [ 100]
BsteCR2 .......... .......A.. .......... .......... A......... .......... .......... .......... .......... ..........  [ 100]
          TAS
BsteCR1 CTTATATGCC TCATTTCTCA TTAAGAGTTA GTTGTGTATT GATTTAGGAA TAGTTCGAGT ATTCATTTTA CCAAAACTAT GAAGTGGTTA GGGTCTGTAT  [ 200]
BsteCR2 .......... .......... .......... .......... .......... .......... .......... .T.....C.. .......... ..........  [ 200]

BsteCR1 ATTCAGTTAA GTCTGGAATA CGGGTATGCC CGAACAAGAG TGAGTGAATG GTGACAGGCC ATAATTACTC ATTAATCCTT CTTGATGTGC CGGTTTCTAA  [ 300]
BsteCR2 .......... .......... .......... .......... .......... .......... .......... .......... .......... ..........  [ 300]
```

```
                                                   F box                                           E box
BsteCR1 AGTATCAGGT GATTTATTAG TCGTTCCCCT CACGAGAAAT CAGCAACCCG GTGTATGTAA GATTTTACGT TACTAGCTTC AGGACCATTC ATTCCCCCTA  [ 400]
BsteCR2 .......... .......... .......... .G......G. .......... .......... .......... .......... .......... ..........  [ 400]
                                                   D box                                            C box
BsteCR1 CACCCTAGCA TAACTTGCGC TTTTGCGCCT CTGGATCCTC GGTCAGGGCC ATGGCTTGGT TTATTTAGTT TTCAGTTCTC TTCACAGAGT CATTTGGTTG  [ 500]
BsteCR2 .......... .......... .......... ....T..... .......... .......... .......... .......... .......... ..........  [ 500]

BsteCR1 ATGCTTGTCT GCTTCTCACC CGTGATCGCG GCATCTGGAT TGCCCGAAGT GCCTCTAGTA TTTTTTCTTT TCTTCGCTTC TTCACAGGTG GCCCTTCGGA  [ 600]
BsteCR2 .......... .......... .......... .......... .......... .......... ........C. ....G..... .......... ..........  [ 600]

BsteCR1 ATGCACCGCG GTGTGGCCAT CGAAGACGTG AGCATACAGA CGCGTCATCG GCCTCTACTA GCTTTCAGGA ATCACTGGAT GAGACGGTTG GAGTATTTGT  [ 700]
BsteCR2 .......... .......... .......... .......... .......... .......... .......... .......... .......... ..........  [ 700]
                         BSB                B box                                            CSB1
BsteCR1 GGAATCATCT TTACCCTGTG CACTTTGTTT TCCATTCAGC TATGGCGTAT CCACTAACCC TAAACATGGT GCTATTTGGT GAATGTTTGC CGGACATAAT  [ 800]
BsteCR2 .......... .......... .......... .......G.. .......... .......... .......... .......... .......... ..........  [ 800]

BsteCR1 CTTACTTACT TCCACCCACT TTTTGCTTCC TCTATCTTTC AACCAAACAG TTAACAAATC TAGGTAATTT TCAACCAAAA ATTAAACAAA CTTTGCAAAA  [ 900]
BsteCR2 .......... .......... .......... .....T.... .......... .......... .......... .......... .......... ..........  [ 900]
                                                              7 x 91 bp + 1 incomplete repeat (85 bp) in CR1
BsteCR1 ACCTTTACAA ACTTTGTTCA TCTACATTCC ATTACTCTAC AACTACTAAA ATCCATTAAA AATTATTTAC CCAATCAGTA TAACTTTTAA CTTATCTTAT  [1000]
BsteCR2 ..T....... .......... .......... .......... .......... .......... .......... .......... .......... ..........  [1000]
                                                              8 x 91 bp + 1 incomplete repeat (90 bp) in CR1
BsteCR1 CTGTATAGTT ATACTCTTTA CTCTATTAAA CACTAGTTTA ATTACATTAA AAATTATTTA CCCAATCAGT ATAACTTTTA ACTTATCTTA TCTGTATAGT  [1100]
BsteCR2 .......... .......... .......... .......... .......... .......... .......... .......C.. .......... ..........  [1100]

BsteCR1 TATACTCTTT ACTCTATTAA ACACTAGTTT AATTACATTA AAAATTATTT ACCCAATCAG TATAACTTTT AACTTATCTT ATCTGTATAG TTATACTCTT  [1200]
BsteCR2 .......... .......... .......... .......... .......... .......... .......... .......... .......... ..........  [1200]

BsteCR1 TACTCTATTA AACACTAGTT TAATTACATT AAAAATTATT TACCCAATCA GTATAACTTT TAACTTATCT TATCTGTATA GTTATACTCT TTACTCTATT  [1300]
BsteCR2 .......... .......... .......... .......... .......... .......... .......... .......... .......... ..........  [1300]

BsteCR1 AAACACTAGT TTAATTACAT TAAAAATTAT TTACCCAATC AGTATAACTT TTAACTTATC TTATCTGTAT AGTTATACTC TTTACTCTAT TAAACACTAG  [1400]
BsteCR2 .......... .......... .......... .......... .......... .......... .......... .......... .......... ..........  [1400]

BsteCR1 TTTAATTACA TTAAAAATTA TTTACCCAAT CAGTATAACT TTTAACTTAT CTTATCCGTA TAGTTATACT CTTTACTCTA TTAAACACTA GTTTAATTAC  [1500]
BsteCR2 .......... .......... .......... .......... .......... ......T... .......... .......... .......... ..........  [1500]

BsteCR1 ATTAAAAATT ATTTACCCAA TCAGTATAAC TTTTAACTTA TCTTATCTGT ATAGTTATAC TCTTTACTCT ATTAAACACT AGTTTAATTA CATTAAAAAT  [1600]
BsteCR2 .......... .......... .......... .......... .......... .......... .......... .......... .......... ..........  [1600]

BsteCR1 TATTTACCCA ATCAGTATAA CTTTTAACTT ATCTTATCTG TATAGTTATA CTCTTTACTC TATTAAACAC TAGTT----- ---------- ----------  [1700]
BsteCR2 .......... .......... .......... .......... .......... .......... .......... .....TAATT ACATTAAAAA TTATTTACCC  [1700]

BsteCR1 ---------- ---------- ---------- ---------- ---------- ---------- ---------- ---------- ---------- ----------  [1800]
BsteCR2 AATCAGTATA ACTTTTAACT TATCTTATCT GTATAGTTAT ACTCTTTACT CTATTAAACG CTAGTTTAAT TACATTAAAA ATTATTTACC CAATCAGTAT  [1800]

BsteCR1 ---------- ---------- ---------- ---------- ---------- ---------- ---------- ---------- ---------- ----------  [1900]
BsteCR2 AACTTTTAAC TTATCTTATC TGTATAGTTA TACTCTTTAC TCTATTAAAC ACTAGTTAAT TCCCTCATCA ACCACACCCT AACATCTCTC TCAACACTAC  [1900]

BsteCR1 ---------- ---------- ---------- ---------- ---------- ---------- ---------- ---------- ---------- ----------  [2000]
BsteCR2 TAATCAACCA TACAAACAAA TAAAACACAA ACAAATAAAA CACAAACAAA TAAAACACAA ACAAATAAAA CACAAACAAA TAAAACACAA ACAAATAAAA  [2000]

BsteCR1 ---------- ---------- ---------- ---------- ---------- ---------- ---------- ---------- ---------- ----------  [2100]
BsteCR2 CACAAACAAA TAAAACACAA ACAAATAAAA CACAAACAAA TAAAACACAA ACAAATAAAA CACAAACAAA TAAAACACAA ACAAATAAAA CACAAACAAA  [2100]

BsteCR1 ---------- ---------- ---------- ---------- ---------- ---------- ---------- ---------- ---------- ------ [2196]
BsteCR2 TAAAACACAA ACAAATAAAA CACAAACAAA TAAAACACAA ACAAATAAAA CACAAACAAA TAAAACACAA ACAAATAAAA CACAAACAAA TAAAAC [2196]


                                                                                                 C stretch
IflaCR1 TTCCATAAAA CCTAATAAAA GCTTAATTAT GTACGGCTAC CATTCCCTGC TCGTTAGACA TCCTAACCCT CATATCCTAC CCCCCCCTTC CCCCCCAAAC  [ 100]
IflaCR2 .......... .......... .......... .......... .......... .......... .......... .......... .......... ..........  [ 100]
           TAS                  TAS                     TAS                              TAS        TAS
IflaCR1 ACGTATATGT CTAGGTAAGT TCTATGTATG AGCATGCATT AAGCTATATG CCCCATTAAC ATTAGTATTA ATGTAAGTAG TATATTAAGT GTATGTGTTA  [ 200]
IflaCR2 .......... .......... .......... .......... .......... .......... .......... .......... .......... ..........  [ 200]

IflaCR1 AGTCCATTTT AAGTGTAAGT ATGGATTAAC CTAGTTTTTC CACTGCTTTG TTTCATATGG ATTAATACTG TAATGAACTT AGGAATAGCT TCATTTAATT  [ 300]
IflaCR2 .......... .......... .......... .......... .......... .......... .......... .......... .......... ..........  [ 300]

IflaCR1 GTACTAAAAC CATGCTCGTA TGAGTCGTAC ATGCTATATG GGGTCGGTGT ACGACTGTGC TTGAGTTAAA GTGGATGAAT GGTAGCAGGC CATAATAGTC  [ 400]
IflaCR2 .......... .......... .....G.... .......... .......... .......... .......... .......... .......... ..........  [ 400]
                                                                          F box
IflaCR1 CCCTTATCTC TTGAAGTACC GGTATCTGAA GTACCAGGTG ATTTATTAGT CGTTCCTCTC ACGTGAAATC AGCAACCCGC CGCATATAAG GCTCTACGTT  [ 500]
IflaCR2 .T........ .......... .......... .......... .....A.... .......... .......... .......... .......... ..........  [ 500]
```

```
         E box                                                                D box
IflaCR1 ACTAGCTTCA GGACCATTCA TTCCCCCTAC ACCCTAGCCC AACTTGCGCT TTTGCGCCTC TGGTTCCTCG GTCAGGGCCA TGGCTCGGTT GACTTAGCAC  [ 600]
IflaCR2 .......... .......... .......... .......... .......... .......... .......... .......... .......... ..........  [ 600]
                  C box
IflaCR1 TGGATGCTCT TCACAGAGTC ATTTGGTTGA TGCTTGTCTG CTTCTCACCC GTGATCGCGG CATCTGGATT GCCTGGGGCG CCTCTAGTAT TTTTTTTATC  [ 700]
IflaCR2 .......... .......... .......... .......... .......... .......... .......... ...C...... .......... ..........  [ 700]

IflaCR1 TTCACTTCTT CACAGGTGGC CCTTCAGAGT GCACCGCGGC GCGGCCATCG AAGACGTGAG CATACAGACG CGTTGCGTTC TTTTATTAGC CTTCAGGAAT  [ 800]
IflaCR2 .......... .......... .......... .......... .......... ........T. .......... .......... .......... ..........  [ 800]
                                                       BSB                 B box
IflaCR1 TACAGGATGA GACGGTTGGA GTATTTGTGG AATCATCTTT ACACTGTGCA CTTTGTTTTC CATTTGGTTG TTGGTGTGTC CACTAACCCC AAACATGGTG  [ 900]
IflaCR2 .......... .......... .......... .......... .......... .......... .......... .......... .......... ..........  [ 900]
                CSB1
IflaCR1 CTATTTGATG AATGCTTGTT GGACATACTT TTACTCGTTT CTACTCATTT ACACTTCCTC TAGTTTTTCT AACAAACGAT AATCAAACTA GGCAAATTTC  [1000]
IflaCR2 .......... .......... .......A.. .......... .......... .......... .......... .......... .......... ..........  [1000]

IflaCR1 AACAAAAAAT TTTGACAAGT CTTATCAAAA ATTTTTACAA ACTTTATTCA TTTGTATTAC ACTATCTTAT ACCACCTTAT AACCACTGGA ATTACATTAA  [1100]
IflaCR2 .......... .......... .......... .......... .......... .......... .......... .......... .......... ..........  [1100]

IflaCR1 AAAATCAAAC CCTAAACAAC CTAAATTTTA ACCCGTGTTA TTTGTATGTT TATATTTTTA TATTTTTTAC CTTCCACACC GCTGAAGTTA CATTAAAAAA  [1200]
IflaCR2 .......... .......... .......... .......... .......... .......... .......... .......... .......... ..........  [1200]

IflaCR1 ATATGCACTA TCATGTTTAT ATCA------ ---------- ---------- ---------- ---------- ---------- ---------- ----------  [1300]
IflaCR2 .......... .......... ....CTACTA CATCTCCCCA GCACAAACAA GCAGCAAACA AGCAGCAAAC AAGCAGCAAA CAAGCAGCAA ACAAGCAGCA  [1300]

IflaCR1 ---------- ---------- ---------- ---------- ---------- ---------- ---------- ---------- ---------- ----------  [1400]
IflaCR2 AACAAGCAGC AAACAAGCAG CAAACAAGCA GCAAACAAGC AGCAAACAAG TAATAAACAA ACAAGTAATA AACAAACAAG TAATAAACAA ACAAGTAATA  [1400]

IflaCR1 ---------- ---------- ---------- ---------- ---------- ---------- ---------- ---------- ---------- ----------  [1500]
IflaCR2 AACAAGCAAG TAATAAACAA ACAAGTAATA AACAAACAAG TAATAAACAA ACAAGTAATA AACAAACAAG TAATAAACAA ACAAGTAATA AACAAACAAG  [1500]

IflaCR1 ---------- ---------- ---------- ---------- ---------- ---------- ---------- ---------- ---------- ----------  [1600]
IflaCR2 TAATAAACAA ACAAGTAATA AACAAACAAG TAATAAACAA ACAAGTAATA AACAAACAAG TAATAAACAA ACAAGTAATA AACAAACAAG TAATAAACAA  [1600]

IflaCR1 ---------- ------ [1616]
IflaCR2 ACAAGTAATA AACAAA [1616]


                                                                C stretch         TAS                  TAS
IeurCR1 TCTTAAAAAC TATGTACGAT TACCATCCCC TCTAATCCAC TGGATACAGC ACCCCCCCCT TCCCCTCCAA ACATATATGT CTAGGTAAGT TCTATGTATA  [ 100]
IeurCR2 .......... .......... .......... .......... .......... .......... .....C.... .......... .......... .........G  [ 100]
                           TAS          TAS                                         TAS
IeurCR1 ACCATGCATT AACTCTTATG TCTTGCTACA TTACAGTCAA TGTTTTGGTA ACATGTTAAA TGTATGTATT AGGTCTGTTT AAGTTTACTA ATAAGGAATA  [ 200]
IeurCR2 .......... ....TAC... C..CAT.... ....---T.. ....GA.TC. GA.AT....G .......... .AAA...--. ....A.TT.. T..T.AC..G  [ 200]
                       TAS          TAS
IeurCR1 -GGTTTGATT TACATACTAT CTTGTTTAAG TATAAGTTAG TGTACTGAAT TAAGGATGAT TTAACACTTG TTAATACTAA AACCATAACT TTTATGGATT  [ 300]
IeurCR2 T.....AG.. .GTTG..AT. TA.A...... .......... .......... .......... .......... .......... .......... ..........  [ 300]

IeurCR1 GTACATGAAT TATGGGGTCG GTGTATGACT ATGTTTGGGT CAAGGTAAAT GAATGGTGAC AGGTCATAAT AATCCAATAA TCTTTTTAGA AGTACCGGTT  [ 400]
IeurCR2 .......... .......... .......... .......... .......... .......... .......... .......... .......... ..........  [ 400]
                                                F box                                             E box
IeurCR1 TCTGAAGTGT TGGGTTATTT ATTAATCGTT CTTCTCACGT GAAATCAGCA ACCCGGTGTA CGTAAGATTT TACGTTACTA GCTTCAGGAC CATTCATTCC  [ 500]
IeurCR2 .......... .......... .......... .......... .......... .......... .......... .......... .......... ..........  [ 500]
                                                   D box                                                 C box
IeurCR1 CCCTACACCC TAGCCCAACT TGCGCTTTTG CGCCTCTGGT TCCTCGGTCA AGGCCATAGC TCGGTTTATT TAGCACTTAG TTCTCTTCAC AGAGTCATTT  [ 600]
IeurCR2 .......... .......... .......... .......... .......... G......... .......... .......... .......... ..........  [ 600]

IeurCR1 GGTTGATGCT TGTCTGCTTC TCACCCGTGA TCGCGGCATC TGGATTGCCC GAAGTGCCTT TAGTAATTTT TTCTTTCTTC ACTTCTTCAC AGGTGGCCCT  [ 700]
IeurCR2 .......... .......... .......... .......... .......... .......... .......... .......... .......... ..........  [ 700]

IeurCR1 TCAGAGTGGA CCGCGGTGCA GCCATCGAAG ACGTGAGCAT ACAGACGCGT TGCGTCCTAT TATTAGCTTT CAAGAATCAC TGGATGAGAC GGTTGAAGTA  [ 800]
IeurCR2 .......... .......... .......... .......... .......... .......... .......... .......... .......... ..........  [ 800]
                                BSB                 B box                                              CSB1
IeurCR1 TTTGTGGAAT CATCTTTACC CTGTGCACTT TGTTTTCCAT TCGGTTGTTG GTGTGACCAC TAACCCTAAA CATGGTGCTA TTTGGTGAAT GATTGCCGGA  [ 900]
IeurCR2 .......... .......... .......... .......... .......... .......... .......... .......... .......... ..........  [ 900]

IeurCR1 CATAATTTTA CTTAATTTTA CCCATTTACA CTTCCTCTAA CTTTCCTACA AAAAATATTT AAACTTAGGT AAATTTCAAC AAAAAAATTT AACACACTCA  [1000]
IeurCR2 .......... .......... .......... .......... .......... .......... .......... .......... .......... ..........  [1000]

IeurCR1 ACAAAAATTT TTACAAACTT TATTCACTTG TATTACACTA TCCTCTAACC ACTAAAATCC CATTAAAAAA TCAAACTCCA AGTAACCCAA TTTTTTAACC  [1100]
IeurCR2 .......... .......... .......... .......... .......... .......... .......... .......... .......... ..........  [1100]

IeurCR1 CATATTTATT TGTATGCTTA TATTTTTTTA CCTTCCACAC CACTGAAGTT ACATTAAAAA ATATATACCT TTTCACTGCT TATGCCATAA CC--------  [1200]
IeurCR2 .......... .......... .......... .......... .......... .......... .......... .......... .......... ..CCACACAA  [1200]
```

```
IeurCR1 ---------- ---------- ---------- ---------- ---------- ---------- ---------- ---------- ---------- ----------  [1300]
IeurCR2 ACGAACACCA AACACAAACG AACACCAAAC ACAAACGAAC ACCAAACACA AACGAACACC AAACACAAAC GAACACCAAA CACAAACGAA CACCAAACAC  [1300]

IeurCR1 ---------- ---------- ---------- ---------- ---------- ---------- ---------- ---------- ---------- ----------  [1400]
IeurCR2 AAACGAACAC CAAACACAAA CGAACACCAA ACACAAATTA GCAACAAACA CAAATTAGCA ACAAACACAA ATTAGCAAAA CACAAATTAG CAAAACACAA  [1400]

IeurCR1 ---------- ---------- ---------- ---------- ---------- ---------- ---------- ---------- ---------- ----------  [1500]
IeurCR2 ATTAGCAAAA CACAAATTAG CAACAAACAC AAATTAGCAA CAAACACAAA TTAGCAACAA ACACAAATTA GCAACAAACA CAAATTAGCA ACAAACACAA  [1500]

IeurCR1 ---------- ---------- ---------- ---------- ---------- ---------- ---------- ---------- ---------- ----------  [1600]
IeurCR2 ATTAGCAACA AACACAAATT AGCAACAAAC ACAAATTAGC AACAAACACA AATTAGCAAC AAACACAAAT TAGCAACAAA CACAAATTAG CAACAAACAC  [1600]

IeurCR1 ---------- ---------- ---------- ---------- ---------- ---------- ---------- ---------- ---------- ----------  [1700]
IeurCR2 AAATTAGCAA CAAACACAAA TTAGCAACAA ACACAAATTA GCAACAAACA CAAATTAGCA ACAAACACAA ATTAACAACA AACACAAATT AGCAACAAAC  [1700]

IeurCR1 ---------- ---------- ---------- ---------- ---------- ---------- ---------- ---------- ---------- ----------  [1800]
IeurCR2 ACAAATTAAC AACAAACACA AATTAGCAAC AAACACAAAT TAACAACAAA CACAAATTAG CAACAAACAC AAATTAGCAA CAAACACAAA TTAACAACAA  [1800]

IeurCR1 ---------- ---------- ---------- ---------- ---------- ------- [1857]
IeurCR2 ACACAAATTA GCAACAAACA CAAATTAACA ACAAACACAA ATTAACAAAA CACAAAT [1857]


                                                                     C stretch           TAS                        TAS
IsinCR1 CCTAAAAAAC TATAAACCCC CCCCAGCACA GACATATAAC CTTAAGCCTA CCCCCCCTAC CCCCCCAGAA CGTATATGTT CTAGGTAAGT TCTATGTATG  [ 100]
IsinCR2 .......... .......... .......... .......... .......... .......... .......... .......... .......... ..........  [ 100]
                          TAS          TAS
IsinCR1 GTCATGCATT AATTTATATG CCCCATTACA TTAAGTTAAT GTTAGATAGC CTATT-AAAT GAATGTGTTG AGTCCATTTA ACTTATAGGT TTAGGATAAT  [ 200]
IsinCR2 .CT....... GG........ .......G.. .C.TA..... .C..AG.T.T AC...T..G. .T....AC.A G.A.T.GG-- ----...AT. ..CAT..GGC  [ 200]

IsinCR1 CTCAGTTATT TACCACTGTT TTGTTTCTAG GGATTAACTC TGTAATGAGT CTAGGAATAG CTTCACTTAC TGTACTAAAA CCATAGTAAT GCTGGTAATG  [ 300]
IsinCR2 A.A.C....A .TTA...CA. ACA.CCT... A......... .......... .......... .......... .......... .......... ..........  [ 300]

IsinCR1 TATAAGGTAT GGGGTCGGTG TATGACTATG CTTGGAACCA AGATAACTGA ATGGTAACAG GTCATGATAG TTCTTCTACT TCTTGTAGTA CCGGTATCTG  [ 400]
IsinCR2 .......... .......... .......... .......... .......... .......... .......... .......... .......... ..........  [ 400]
                                        F box                                               E box
IsinCR1 AAGTACCAGG TGATTTATTA GTCGTTCTCC TCACGTGAAA TCAGCAACCC GGTGCACATA AGGTTTTACG TTACTAGCTT CAGGACCATT CTTTCCCCCT  [ 500]
IsinCR2 .......... .......... .......... .......... .......... .......... .......... .......... ........C. ..........  [ 500]
                                          D box                                                    C box
IsinCR1 ACACCCTAGC ACAACTTGCG CTTTTGCGCC TCTGGTTCCT CGGTCAGGGC CATAGCTCGG TTTACTTAGC ACTTAATGCT CTTCACAGAG TCATTTGGTT  [ 600]
IsinCR2 .......... .......... .......... .......... .......... .......... .......... .......... .......... ..........  [ 600]

IsinCR1 GATGCTTGTC TGCTTCTCAC CCGTGATCGG GGCATCTGGA TTGCCTGGGG CGCCTCTAGT ATTTTTTTCT TCTTCACTTC TTCACAGGTG GCCCCTCAGA  [ 700]
IsinCR2 .......... .......... .........C .......... .......... .......... .......... .......... .....G.... ..........  [ 700]

IsinCR1 GTGCACCGCG GCGCAGCCAT CGAAGACGTG AGCATACAGA CGCGTTGCGT CCTATTATTA GCTTTCAAGA ATCACTGGAT GAGACGGTTG AAGTATTTGT  [ 800]
IsinCR2 .......... .......... .......... .......... .......... .......... .......... .......... .......... ..........  [ 800]
                           BSB                 B box                                          CSB1
IsinCR1 GGAATCATCT TTACCCTGTG CACTTTGTTT TCCATTTGGT TGTTGGTGTG ACCACTAACT CTAAACATGG TGCTATTTGG TGAATGTTTG CTGGACATAA  [ 900]
IsinCR2 .......... .......... .......... .......... .......... .......... ....G..... .......... .......... ..........  [ 900]

IsinCR1 TTTTACTTAC TTTCACTCAT TTTCATTTCC TCTATTTTCT TAACAAACTT TCACAAACCT AGGAAAATTT TAACCAAAAA TTTAACAAGC TTCATTAAAA  [1000]
IsinCR2 .......... .......... .......... .......... .......... .A........ .......... .......... .......... ..........  [1000]
                          2 x 92 bp + 1 incomplete repeat (78 bp)
IsinCR1 ATTTTTACAA ACTTTGTTTA CTTATATTAC ACTATCTTTA ACCACTGAAA TTTCATTAAA ATCAAACCTT AGTTAACACA AACTTTTATC AAAAATTTTT  [1100]
IsinCR2 .......... .......... .......... .......... .......... .......... .......... .......... .......... ..........  [1100]

IsinCR1 AAACTTTGTT TACTTATATT ACACTATCTT TAACCACTGA AATTTCATTA AAATCAAACC TTAGTTAACA CAAACTTTTA TCAAAAATTT TTAAACTTTG  [1200]
IsinCR2 .......... .......... .......... .......... .......... .......... .......... .......... .......... ..........  [1200]

IsinCR1 TTTACTTATA TTACACTATC TTTAACCACT GAAATTTCAT TAAAATCAAA CCTTAGTTAA CACAAACTTT ATCCGTGTTA TTTGTATGTT GTATATTCGT  [1300]
IsinCR2 .......... .......... .......... .......... .......... .......... .......... .......... .......... ..........  [1300]

IsinCR1 ATATTTTTTA CCCTTCAAAC CGCTGGAGTT ACATTGAAAA AATATA---- ---------- ---------- ---------- ---------- ----------  [1400]
IsinCR2 .......... .......... .......... .....A.... ......TGCC ATCATATGGT CATCACAACT CATTACGCCA AATAAGCAAC AAACACAAAT  [1400]

IsinCR1 ---------- ---------- ---------- ---------- ---------- ---------- ---------- ---------- ---------- ----------  [1500]
IsinCR2 AAGCAACAAA CACAAATAAG CAACAAACAC AAATAAGCAA CAAACACAAA TAAGCAACAA ACACAAATAA GCAACAAACA CAAATAAGCA ACAAACACAA  [1500]

IsinCR1 ---------- ---------- ---------- ---------- ---------- ---------- ---------- ---------- ---------- ----------  [1600]
IsinCR2 ATAAGCAACA AACACAAATA AGCAACAAAC ACAAATAAGC AACAAACACA AATAAGCAAC AAACACAAAT AAGCAACAAA CACAAATAAG CAACAAACAC  [1600]

IsinCR1 ---------- ---------- ---------- ---------- ---------- ---------- ------ [1666]
IsinCR2 AAATAAGCAA CAAACACAAA TAAGCAACAA ACACAAATAA GCAACAAACA CAAATAAGCA ACAAAC [1666]
```

C stretch
IcinCR1 ---------- ---------- ---------- ---------- ---------- ---------- ---------- ---------- ---ACCCCCC CCTACCCCCC [100]
IcinCR2 TTTCAAACCT TCCCCAAAAA ATCATGTACA ACTACCATTT CTTAATCCCC TAGACATAAT ACTCAATCCC CCAATCTACT CGC.-..... .T........ [100]
TAS TAS TAS TAS TAS
IcinCR1 CAAACATATA TGTCTGGGTA AATTTTATGT ATGACCATGC ATTAACTTAT ATGCCTCATT ACATTGCAGT TAATGTTGAG TAGCATATTA AAT-GTATGT [200]
IcinCR2 -......... ...TCA.... .G..AC.... ..AG.T.... .....T...C .... --...AT.-. ....A.A .CATGGG..T ...T...... [200]

IcinCR1 GTTAGGTCTA TTTGAATTTA TAGATAAGGA TTAAGTTTGG TTTGTTTACT ATTTTGTTTA AGTATATTAT CATGTATTAA ACCAGGAATA GTTTCTTTTA [300]
IcinCR2 AC....A... .GGA.G..CT .---..T.AT A..TAA..T. ..AA..C.T. --........ C..G..A... AT......G. ..T.AC.... AC...AC.C. [300]

IcinCR1 GTTAGTACTA AAACCATAAG ATTCATGGGG TGTACATACT TTATGGGGTC AGTATACGGC TATGTTTTGG TTAAGGTATC TGAATGGTAA CAAGCCATGA [400]
IcinCR2 -C..A..... ....A. ....A. [400]
F box
IcinCR1 TAATTCAATT ATCTCTTGAA GTACCGGTGT CTGAAGTGTT AGGTTATTTA TTAATCGTTC TTCTCACGTG AAATCAGCAA CCCGGTGTAC GTAATGTTTT [500]
IcinCR2 .... [500]
E box D box
IcinCR1 ACGTTACTAG CTTCAGGACC ATTCATTCCC CCTACACCCT AGCCCAACTT GCGCTTTTGC GCCTCTGGTT CCTCGGTCAG GGCCATGGCT CGGTTTACTT [600]
IcinCR2 .... [600]
C box
IcinCR1 AGCACTCGGT CCTCTTCACA GAGTCATTTG GTTGATGCTT GTCTGCTTCT CACCCGTGAT CGCGGCATCT GATTGCCCGA AGTGCCTCTA GTAATTTTTT [700]
IcinCR2 .... [700]

IcinCR1 TTTCTTCACT TCTTCACAGG TGGCCCCTCA GAGTGCACCG CGGTGCAGCC ATCGAAGACG TGAGCATACA GACGCGTTGC GTCCTATTAT TAGCTTTCAA [800]
IcinCR2 .... [800]
BSB B box
IcinCR1 GAATCACTGG ATGAGACGGT TGAAGTATTT GGGGAATCAT CTTTACCCTG TGCACTTTGT TTTCCATTTG GTTGTGGTGG GACCGCTAAC CCTAAACATG [900]
IcinCR2 ....G...... .... .C........ .... [900]
CSB1
IcinCR1 GTGCTATTTG GTGAATGTTT GCCGGACATA ATTTCACTTA CTTTTACCCA TTTACACTTC CTCTAACTTT TCTAACAAAC TATTTTTAAA CTAGGTAAAT [1000]
IcinCR2 .... [1000]

IcinCR1 TTCAACAAAA AATTTAACAA ACCTTAACAA AAATTTTTAC AAACTTTGTT AATTTGTATT ACACTACTCC ATAACCAGCA GGATCCCATT AAAAACCAAA [1100]
IcinCR2 .... .A........ .... [1100]

IcinCR1 CCCTTTAACA ACCTAATTTT TTTTAGCCCA TATCTATTTG TATGCTTATA TTTTTCATAT ACTACACCAC TGGAGTTACA TTAAAAAAAT ACTGATCATC [1200]
IcinCR2 ....A.... .... [1200]

IcinCR1 GAGCGTACAC CATAACTC-- ---------- ---------- ---------- ---------- ---------- ---------- ---------- ---------- [1300]
IcinCR2 ....CA CACAACACCA TAACTTCACA CCAAATAAGC AATAAACACA AATAAGCAAT AAACACAAAT AAGCAATAAA CACAAATAAG [1300]

IcinCR1 ---------- ---------- ---------- ---------- ---------- ---------- ---------- ---------- ---------- ---------- [1400]
IcinCR2 CAATAAACAC AAATAAGCAA TAAACACAAA TAAGCAATAA ACACAAATAA GCAATAAACA CAAATAAGCA ATAAACACAA ATAAGCAATA AACACAAATA [1400]

IcinCR1 ---------- ---------- ---------- ---------- ---------- ---------- ---------- ---------- ---------- ---------- [1500]
IcinCR2 AGCAATAAAC ACAAATAAGC AATAAACACA AATAAGCAAT AAACACAAAT AAGCAATAAA CACAAATAAG CAATAAACAC AAATAAGCAA TAAATACAAA [1500]

IcinCR1 ---------- ---------- ---------- ---------- ---------- ---------- ---------- ---------- ---------- ---------- [1600]
IcinCR2 TAAGCAATAA ACACAAATAA GCAATAAACA CAAATAAGCA ATAAACACAA ATAAGCAATA AACACAAATA AGCAATAAAC ACAAATAAGC AATAAACACA [1600]

IcinCR1 ---------- ---------- ---------- ---------- ---------- ---------- ---------- [1670]
IcinCR2 AATAAGCAAT AAACACAAAT AAGCAATAAA CACAAATAAG CAATAAACAC AAATAAGCAA TAAACACAAT [1670]

C stretch TAS
AcinCR1 ACCCACTAAC AGCATCACAG TGGCCCTATA CATGGACCCC CCCC-TTCCC CCCCCATACG CATATGCTCA GGCGAGTCGT ATGTATGGGC ATGCATTGGT [100]
AcinCR2 ....C..-.. ....T....TA. G.A.G.ATTA GCA...-.AC [100]
TAS TAS TAS
AcinCR1 CTATATGCCC CATGCATTGT ATCAATGTTA GAGAATGCAG TTATATGCAT GTACTAGTTC CATGCTATGT CTT---CAGT ACAAGTTCTT GATTGTCCAC [200]
AcinCR2 .....CATT. T.C.TG.CAA GCT....... A.TC.CA..T .A..C..T.. ....G..G.. ...AAA...C T.GATAT.A. .TTTA..... A..C.G...T [200]

AcinCR1 CTGTCTTATC TTAGAGGATT AATCTTGTAT TGAGCTTAGG AATGGCTTCA TAATCTGTAC TAAAACCATA TACAGTAATG GGCCGTACAT GTACTATGGG [300]
AcinCR2 AA..-....- .C........ ....T...... .... [300]

AcinCR1 GTCAAGTGTA CGGCTGTGCT TGAATTCAGT TGATTGAATG GTGACAGGTC ATGGTAGTTC AATAGTCTCT TGGGGTGCCG GTATCTGAAG TACCAGGTTA [400]
AcinCR2 ....A. .... [400]
F box E box
AcinCR1 TTTATTGGTC GTTCTTCTCA CGTGAAATCA GCAACCCGCC GCATAGAAGG CTCTACGTTA CTAGCTTCAG GACCATTCTT TCCCCCTACA CCCTAGCGCG [500]
AcinCR2 .... [500]
D box C box
AcinCR1 ACTTGCTCTT TTGCGCCTCT GGTTCCTCGG TCAGGGCCAT GGCTCGGTTG ACTTAGCACT CGGTCCTCTT CACAGAGTCA TTTGGTTGAT GCTTGTCTGC [600]
AcinCR2 .... [600]

AcinCR1 TTCTCACCCG TGATCGCGGC ATCTGGATTG CCTGGGGCGC CTCTAGTATT TTTCTCTTCT AAACTTCTTC AGGCTGCCCT CCGGTGCACC GCGGCGCAGC [700]
AcinCR2 .... [700]

```
AcinCR1 CATCGAAGAC GTGAGCATAC AGACGCGTCA TCGGCCTCTT ATTAGCACTC AGGAATGACT GGATGAGACG GTTGGAGTAT TTGGGGAATC ATTTTTACAC  [ 800]
AcinCR2 .......... .......... .......... .......... .......... .......... .......... .......... .......... ..........  [ 800]
           BSB                  B box                                           CSB1
AcinCR1 TGTGCACTTT GTTTTCCATT TGGTTGTTGG TGTGTCCACT AACCCCTAAC ATGGTGTTAT TTGTTGAATG CTTGTTGGAC ATAATTTTAT TTCTTTTCTT  [ 900]
AcinCR2 .......... .......... .......... .......... .......... .......... .......... .......... .......... ..........  [ 900]

AcinCR1 CTTGTTTACA CTTCCTCTAA TTTTCATTCA CTCTATAAAT CAAGCTAGGT AAATTTCAGC TAAAAATTTA ACAAGCCTTA TTAAAAATTT TTCACGAATC  [1000]
AcinCR2 .......... .......... .......... .......... .......... .......... .......... .......... .......... ..........  [1000]

AcinCR1 TTATTCTTAT ATTTTACATT TCCTTCAACC ACTGAAGTTC CATTAAAAAA TACACCCAAA TCAACGTAAC TTTTTTCGGT GTGTTATTAT ATATTTACAC  [1100]
AcinCR2 .......... .......... .......... .......... .......... .......... .......... .......... .......... ..........  [1100]

AcinCR1 ATAATTATTA CCCTCCACAC CGCTGAAGTT ACATTAAAAA AATAAAGCAT TATTATGCTT TACATAACTA AATTTCATGC CCT------- ----------  [1200]
AcinCR2 .......... .......... .......... .......... .......... .......... .......... .......... ...CTTTCCT ACCAAACACT  [1200]

AcinCR1 ---------- ---------- ---------- ---------- ---------- ---------- ---------- ---------- ---------- ----------  [1300]
AcinCR2 ACTAAAATTT CAAACAAACG ATCAAACGAT CAAACGATCA AACGATCAAA CGATCAAACG ATCAAACGAT CAAACGATCA AACGATCAAA CGATCAAACG  [1300]

AcinCR1 ---------- ---------- ---------- ---------- ---------- ---------- ---------- ---------- ---------- ----------  [1400]
AcinCR2 ATCTAAACAA CAAACAACAA ACAACAAACA ACAAACAACA AACAACAAAC AACAAACAAC AAACAACAAA CAACAAACAA CAAACAACAA ACAACAAACA  [1400]

AcinCR1 ---------- ---------- ---------- ---------- ---------- ---------- ---------- ---------- ---------- ----------  [1500]
AcinCR2 ACAAACAACA AACAACAAAC AACAAACAAC AAACAACAAA CAACAAACAA CAAACAACAA ACAACAAACA ACAAACAACA AACAACAAAC AACAACAAAC  [1500]

AcinCR1 --- [1503]
AcinCR2 ACT [1503]


                                       C stretch        TAS                             TAS           TAS          TAS
ApurCR1 GCCCATAACA GCATCACAGA CCCCCCCCCT TCCCCCCCCA TACATTTATG CTTAGGCAAG CTGTATGTAT GGGCATGCAT TGGTCTATAT GCCCCATGCA  [ 100]
ApurCR2 .......... .......... .......... .......... .....GC... ....A.T..A TCA....... ..TTG.A... CA.C....C. ..TT....T.  [ 100]
                                 TAS              TAS
ApurCR1 TTCTGT--TA ATGTTAGATA ATACAGTTAT AT-------G CATGTACTAG TTCTATTTTA TGTCCTCAGT GCAAGTTCTT GATTGTCCAC CTGTCTTATC  [ 200]
ApurCR2 ..A...AT.. .ATC..T..T .A..T..C.. .CCAATTAC. T...C..G.A G..C..AAA. ...TTGAGA. AAT.A..A.. CT.AA..GG. .ATAGA.TA.  [ 200]

ApurCR1 TTAGAGGATT AATCTTGTAT TGAGTTTAGG AATAGCTTCA TAATCTGTAC TAAAACCATA TACAGTAGTG GGCTGTACAT AATCTATGGA TTAGGCGTAT  [ 300]
ApurCR2 .C........ .......... .......... .......... .......... .......... .......... .......... .......... ..........  [ 300]

ApurCR1 GGCTGTGCTT GAATCCAGTA AGTGAATGGT GACAGGTCAT GACTGTTCAA CTATCTCTTG AAGTACCGGT ATCTGAAGTA CCAGGTTATT TATTGGTCGT  [ 400]
ApurCR2 .......... .......... .......... .......... .......... .......... .......... .......... .......... ..........  [ 400]
                  F box                                    E box
ApurCR1 TCTTCTCTCG TGAAATCAGC AACCCGCCGC ATATAAGGCT CTACGTTACT AGCTTCAGGA CCATTCATTC CCCCTACACC CTAGCACGAC TTGCTCTTTT  [ 500]
ApurCR2 .......A.. .......... .......... .......... .......... .......... .......... .......... .......... ........C.  [ 500]
                      D box                                                          C box
ApurCR1 GCGCCTCTGG TTCCTCGGTC AGGGCCATGG CTCGGTTGAC TTAGCACTCG GTCCTCTTCA CAGAGTCATT TGGTTGATGC TTGTCTGCTT CTCACCCGTG  [ 600]
ApurCR2 .......... .......... .......... .......... .......... .......... .......... .......... .......... ..........  [ 600]

ApurCR1 ATCGCGGCAT CTGGATTGCC TGGGGCGCCT CTAGTATTTT TTTCTTCTAA ACTTCTTCAG GCTGCCCTCC GGTGCACCGC GGCGCAGCCA TCGAAGACGT  [ 700]
ApurCR2 .......... .......... .......... .......... .......... .......... .......... .......... .......... ..........  [ 700]
                                                                                                          BSB
ApurCR1 GAGCATACAG ACGCGTCATC GGCCTCTTAT AGCACTCAGG AATGACTGGA TGAGACGGTT GGAGTATTTG GGGAATCATT TTTACACTGT GCACTTTGTT  [ 800]
ApurCR2 .......... .......... .......... .......... .......... .......... .......... .......... ..C....... ..........  [ 800]
                 B box                                                  CSB1
ApurCR1 TTCCATTTGG TTGTTGGTGT GTCCACTAAC CCCTAAATAT GCTGCTATTT GTTGAATGCT TGTTGGACAT AATTTTATTT CTTTTCTACT TGTTTACACT  [ 900]
ApurCR2 .......... .......... .......... .........C .......... .......... .......... .......... .......... ..........  [ 900]

ApurCR1 TCCTCTAATT TTCTTTCACT TTACAAACTA GACTAGGTAA ATTTCAACTA AAAATTTAAC AAACCTTATC AAAAATTTTT CACAAATCTT ATTCTTATAT  [1000]
ApurCR2 .......... .......... .......... .......... .......... .......... .......... .......... .......... ..........  [1000]

ApurCR1 TTTACATTTT ACTTACCCGC TGGAGTTCCA TTAAAAAACA TACCCTAAAC AGCGTAACTT TTTTCGGTGT GTTATTATAT ATTTACACAC AATTATTACC  [1100]
ApurCR2 .......... .......... .......... .......... .......... .......... .......... .......... .......... ..........  [1100]

ApurCR1 CTCCGCACTG CTGGAGTTAC ATTAAAAAAA ATAGGCATTA TTATGCTTTA CGTAACTAAA TTCCTTGTCC ---------- ---------- ----------  [1200]
ApurCR2 .......... .......... .......G.. .......... .......... .......... ..T....... ATTTCCCTAC CAAACACCAC TAAAACTTCA  [1200]

ApurCR1 ---------- ---------- ---------- ---------- ---------- ---------- ---------- ---------- ---------- ----------  [1300]
ApurCR2 AACGAGCAAC GAACAACGAA CAACGAACAA CGAACAACGA ACAACGAACA ACGAACAACG AACAACGAAC AACGAACAAC GAACAACGAA CAACGAACAA  [1300]

ApurCR1 ---------- ---------- ---------- ---------- ---------- ---------- ---------- ---------- ---------- ----------  [1400]
ApurCR2 CGAACAACGA ACAACGAACA ACGAACAACG AACAACGAAC AACGAACAGC GAACAACGAA CAATAAACAA ACAATAAACA AACAATAAAC AAACAATAAA  [1400]

ApurCR1 ---------- ---------- ---------- ---------- ---------- ---------- ---------- ---------- ---------- ----------  [1500]
ApurCR2 CAAACAATAA ACAAACAATA AACAAACAAT AAACAAACAA TAAACAAACA ATAAACAAAC AATAAACAAA CAATAAACAA ACAATAAACA AACAATAAAC  [1500]
```

```
ApurCR1 ---------- ---------- ---------- ---------- --- [1543]
ApurCR2 AAACAATAAA CAAACAATAA ACAAACAATA AACAACGAAA TAA [1543]


                                       C stretch            TAS                     TAS         TAS
AintCR1 ---------- ---------- ----CCTCCC CCCTTCCCCC CCCGTAACAT ACATGCTTAG GCAAGTCGTA TGTACGGCCA TGCAT----- ----TGGTCT  [ 100]
AintCR2 ACATCTACAC GACCCTTACT TTCC..C... ....A..... ...A..-... ......C..A .......C.. .....AA.TG .A...CCCAC TCAT.A...A  [ 100]
                   TAS                                TAS      TAS
AintCR1 ATAT-GCCCC ATGTATTGCG TAATGTTAGA TTATACAATT ATATGCATGT ACTATCTTCA TATCGTATTT TGAGTACAA- ----GTTCTT AACTGTATAC  [ 200]
AintCR2 G.G.TAAA.T .CA....TA. .T..A.G..- C.GC.T.T.. .ACC.T.... ..G.GG.C.. .-.AA...G. CT....T..T ATTCA..... ..TC.ACC.-  [ 200]

AintCR1 CTGTCTTATC TTAGGGGATT AATCCTGTAC TGAGTTTAGG AATAGCTCCA TAATCTGTAC TAAAACCATA AGTAACAGTG GGTTGTACAT AAGCTATGGA  [ 300]
AintCR2 -.AGA....- .C........ .......... .......... .......... .......... .......... .......... .......... ..........  [ 300]

AintCR1 TTAAGCGTAT GGCTGTGCTT GAATTCAGTT GTCTGAATGG TGACAGGTCA TGCTAGTTCA ATTATCTCTC GAAGTACCGG TATCTGAAGT ACCAGGTTAT  [ 400]
AintCR2 .......... .......... .......... .......... .......... .......... .......... .......... .......... ..........  [ 400]
                              F box                                         E box
AintCR1 TTATTGGTCG TTCTTCTCAC GTGAAATCAG CAACCCGCCG CATATAAGGC TCTACGTTAC TAGCTTCAGG ACCATTCATT CCCCCTACAC CCTAGCCCAA  [ 500]
AintCR2 .......... .......... .......... .......... .......... .......... .......... .......... .......... ..........  [ 500]
                              D box                                                  C box
AintCR1 CTTGCGCTTT TGCGCCTCTG GTTCCTCGGT CAGGGCCATG GCTCGGTTGA CTTAGCACTT AGTGCTCTTC ACAGAGTCAT TTGGTTGATG CTTGTCTGCT  [ 600]
AintCR2 .......... .......... .......... .......... .......... .......... .......... .......... .......... ..........  [ 600]

AintCR1 TCTCACCCGT GATCGCGGCA TCTGGATTGC CTGGGGCGCC TCTAGTATTT TTTTCTTCTA AACTCCTTCA GGCTGCCCTC CGGTGCACCG CGGCGCAGCC  [ 700]
AintCR2 .......... .......... .......... .......... .......... .......... ....T..... .......... .......... ..........  [ 700]

AintCR1 ATCGAAGACG TGAGCATACA GACGCGTCAT CGGACTCTTA TTAGCGCTCA GGAGTGACTG GATGAGACGG TTGGAGTATT TGGGGAATCA TCTTTACACT  [ 800]
AintCR2 .......... .......... .......... .......... .......... .......... .......... .......... .......... .T........  [ 800]
          BSB              B box                                                 CSB1
AintCR1 GTGCACTTTG TTTTCCACTT GGTTGTTGGT GTGTCCACTA ACCCCTAAAT ATGCTGCTAT TTGTTGAATG CTTGTTGGAC ATAATTTTAT CTCTATTCCT  [ 900]
AintCR2 .......... .......T.. .......... .......... .......... .......... .......... .......... .......... ..........  [ 900]

AintCR1 CTTATTTACA CTTCCTCTAA TTTTCTTTCA TTTTCTAAAC TAATCTAGGG AACTTTCAAC TAAAAATTTA ACGAACTTTA TCAAAAATTT TTCACAAATC  [1000]
AintCR2 .......... .......... .......... .......... .......... .......... .......... .......... .......... ..........  [1000]

AintCR1 TTATTCTTAA ATCTTACATC ACCTTTAACC ACTGGAGTTA CATTAAAAAA CATACCCTAA ACAACGAAAC TTTTTTCGGT GTGTTATTAT ATATTTACAC  [1100]
AintCR2 .......... .......... .......... .......... .......... .......... .......... .......... .......... ..........  [1100]

AintCR1 ATAATTATTA CCCTCCACAC TGCTGAAGTT ACATTAAAAA AAACAAGCAT TATTTATGCT TTGCATAACT AAATTTCTTA TC-------- ----------  [1200]
AintCR2 .......... .......... .......... .......... ..-....... .......... .......... .......... ..TATTACAT CAAACAGCAC  [1200]

AintCR1 ---------- ---------- ---------- ---------- ---------- ---------- ---------- ---------- ---------- ----------  [1300]
AintCR2 TAAAATTTCA AACAAACAAC AAACAACAAA CGATAACAAA CAACAAACGA TAACAAACTA CAAACGATAA CAAACAACAA ACGATAACAA ACAACAAACG  [1300]

AintCR1 ---------- ---------- ---------- ---------- ---------- ---------- ---------- ---------- ---------- ----------  [1400]
AintCR2 ATAACAAACA ACAAACGATA ACAAACAACA AACAACAAAC GATAACAAAC AACAAACGAT AACAAACAAC AAACGATAAC AAACAACAAA CAACAAACGA  [1400]

AintCR1 ---------- ---------- ---------- ---------- ----- [1445]
AintCR2 TAACAAACAA CAAACGATAA CAAACACCAT AAAACACTTA ACACT [1445]


                                       C stretch                                  TAS         TAS         TAS
AmodCR1 ACCCCCCAGC ACCCCATAAT AGGACCCCCC CCCTTCCCCC CCGTACGCAG ACATGCTCAG GCAAGTTCTA TGTACGGCCA TGCATTGGTC TATATGCCCC  [ 100]
AmodCR2 .-...A...G .......... .......... ..-....... ..A..AAT.C ....A..... .......A.. ....T.ATT. .A...----- -------.TT  [ 100]
                         TAS                TAS    TAS
AmodCR1 ATGCATTGCG TTAATGCTAG ATAATACAAT TATATGCATG TACTAGTTTC ATAGTATGTT TTAAGTATAA GTTTTTAATT GTATACC-TG TCTTATCTTA  [ 200]
AmodCR2 G.A..---.. .--..A...A ....C.T.T. ..CC..T... ...G..G.C. ..TAA...C. .G.TA..AT. A..A..CT.A A.CGG..A.A AG....T.C.  [ 200]

AmodCR1 GAGGATTAAT CTTGTATTGA GTTTAGGAAT AGCTTCATAA CCTGTACTAA AACCATATAT AATGGTGGGT TGTACATAAG CTCTGGATAC TGGATACGGC  [ 300]
AmodCR2 .......... .......... .......... .......... .......... .......... .......... .......... .......... ..........  [ 300]

AmodCR1 TGTGCTTGAA TTCAGTTGAT TGAATGGTGG CAGGCCATGC TAGTTCAACT ATCTCTTGGA GTGCCGGTTT CTGAAGTATC AGGTGATTTA TTAGTCGTTC  [ 400]
AmodCR2 .....C.... .......... .......... .......... .......... .......... .......... .......... .......... ..........  [ 400]
                    F box                                      E box
AmodCR1 CTCTCACGTG AAATCAGCAA CCCGCCGCAT ATAAGGCTCT ACGTTACTAG CTTCAGGACC ATTCTTTCCC CCTACACCCT AGCACAACTT GCGCTTTTGC  [ 500]
AmodCR2 .......... .......... .......... .......... .......... .......... .......... .......... .......... ..........  [ 500]
                      D box                                             C box
AmodCR1 GCCTCTGGTT CTTCGGTCAG GGCCATAGCT CGGTGGATTT AGCACTCGGT CCTCTTCACA GAGTCATTTG GTTGATGCTT GTCTGCTTCT CACCCGTGAT  [ 600]
AmodCR2 .......... .......... .......... .......... .......... .......... .......... .......... .......... ..........  [ 600]

AmodCR1 CGCGGCATCT GGATTGCCTG GGGCGCCTCT AGTATTTTTT TCTTCTAAAC TTCTTCAGGC TGCCCTCCGG TGCACCGCGG CGTGGCCATC GAAGACGTGA  [ 700]
AmodCR2 .......... .......... .......... .......... .......... .......... .......... .......... .......... ..........  [ 700]
```

```
                                                                                              BSB
AmodCR1 GCATACAGAC GCGTCATCGG TCTCTTATAG CGCTGGGTAC AACTGGATGA GACGGTTGGA GTATTTGGGG AATCATTTTT ACACTGTGCA CTTTGTTTTC  [ 800]
AmodCR2 .......... .......... .......... .......... .......... .......... .......... .......... .......... ..........  [ 800]
           B box                                        CSB1
AmodCR1 CATTTGGTTG TTGGTGTGTC CACTAACCTT TAGATATGTT GTTATTTGTT GAATGCTTGC TAGACATAAT TTTATCTCTA TTCTTCTTAT TTACACTTCC  [ 900]
AmodCR2 .......... .......... .......... .......... .......... .......... .......... .......... .......... ..........  [ 900]

AmodCR1 TCTAATTTTC TTTAACTTTC TAAATCAAAC CAGGTAACTT TCAACTAAAA ATTTAACAAA CCTTGACAAA AATTTTTCAC AAATCTTATT CTTATATTTT  [1000]
AmodCR2 .......... .......... .......... T......... .......... .......... .....T.... .......... .......... ..........  [1000]
                                     3 x 91 bp + 1 incomplete repeat (21 bp)
AmodCR1 ACATTACCTT TAACCGCTAA AGTTACATTA AAAAATATAC CCTAAACAAC GTAACTTTTT TCGGTGTGTT ATTATATATT TACACATAAT TATTACCCTC  [1100]
AmodCR2 .......... .......... .......... .......... ........G. ......C... .......... .......... .......... ..........  [1100]

AmodCR1 CACACCACTA AAGTTACATT AAAAAATATA CCCTAAACAA CGTAACTTTT TTCGGTGTGT TATTATATAT TTACACATAA TTATTACCCT CCACACCACT  [1200]
AmodCR2 .......... .......... .......... .........G .......... .......... .......... .......... .......... ..........  [1200]

AmodCR1 AAAGTTACAT TAAAAAATAT ACCCTAAACA ACGTAACTTT TTTCGGTGTG TTATTATATA TTTACACGTA ATTATTACCC TCCACACCGC TAGAGTTACA  [1300]
AmodCR2 .......... .......... .......... .......... .......... .......... .......A.. .......... .......... ..........  [1300]

AmodCR1 TTAAAAAAAA CAAACATTAT TATGCTTTAC GTAGCTAAAT TTCATGCC-- ---------- ---------- ---------- ---------- ----------  [1400]
AmodCR2 .......... .......... .......... .......... ........TC TTGTACCAAA CAGCACTAAA ATTTCAAACA AACAACGAAT GATCAACGAA  [1400]

AmodCR1 ---------- ---------- ---------- ---------- ---------- ---------- ---------- ---------- ---------- ----------  [1500]
AmodCR2 CAATCAACGA ATCTGAACGA AACAAACAAA TGAACGATAA CAAACAAACA AATGAACGAT AACAAACAAA CAAATGAACG ATAACAAACA AACAAATGAA  [1500]

AmodCR1 ---------- ---------- ---------- ---------- ---------- ---------- ---------- ---------- ---------- ----------  [1600]
AmodCR2 CGATAACAAA CAAACAAATG AACGATAACA AACAAACAAA TGAACGATAA CAAACAAACA AATGAACGAT AACAAACAAA CAAATGAACG ATAACAAACA  [1600]

AmodCR1 ---------- ---------- ---------- ---------- ---------- ---------- ---------- ---------- ---------- ----------  [1700]
AmodCR2 AACAAATGAA CGATAACAAA CAAACAAATG AACGATAACA AACAAACAAA TGAACGATAA CAAACAAACA AATGAACGAT AACAAACAAA CAAATGAACG  [1700]

AmodCR1 ---------- ---------- ---------- ---------- ---------- ---------- ---------- ---------- ---------- ----------  [1800]
AmodCR2 ATAACAAACA AACAAATGAA CGATAACAAA CAAACAAATG AACGATAACA AACAAACAAA TGAACGATAA CAAACAAACA AATGAACGAT AACAAACAAA  [1800]

AmodCR1 ---------- ---------- ---------- ---------- ---------- ---------- ---------- ---------- ---------- ----------  [1900]
AmodCR2 CAAATGAACG ATAACAAACA AACAAATGAA CGATAACAAA CAAACAAATG AACGATAACA AACAAACAAA TGAACGATAA CAAACAAACA AATGAACGAT  [1900]

AmodCR1 ---------- ---------- ---------- ---------- ---------- ---------- ---------- ---------- ---------- ----------  [2000]
AmodCR2 AACAAACAAA CAAATGAACG ATAACAAACA AACAAATGAA CGATAACAAA CAAACAAATG AACGATAACA AACAAACAAA TGAACGATAA CAAACAAACA  [2000]

AmodCR1 ---------- ---------- ---------- ---------- ---------- -- [2052]
AmodCR2 AATGAACGAT AACAAACAAA CAAATGAACG ATAACAAACA AATCTAAACA CT [2052]


                                   C stretch                                  TAS                    TAS
AibiCR1 ---------- --------AT AGAACCCCCC CCCCT-CCCC CCCATACACA CATGCTCAGG CAAGTTGTAT GTATGGCCAT GCATTGGTCT A-TATGCCCC  [ 100]
AibiCR2 ACCCACATGA ACCCCCAT.. ..G....... ..-T.C.... .......... .......... ......A... ....AA.T.- -....AA.TG .A..CA.T..  [ 100]
                                       TAS
AibiCR1 ATGCATTGTA TTAATGTTAG ATAATACAGT TATATGTATG TACTAATTCC ATGTTATGTG CTG---AGTA CAAGTTTTTG ATTGTCCACC CGTCTTATCT  [ 200]
AibiCR2 ..A..C.AAG .C.T.AC..A .CT.C...T. ...T.A.... ...G.GG... ..AAG....T TAATAT.A.. T.TA..C..A ..C.G...TA GA.TA.---.  [ 200]

AibiCR1 TAGAGGATTA ATTCTGTACT GAGTTTAGGA ATAGCTCCGT AATCTGTACT AAAACCATAT TTAGCAGTGG ACTGTACATA AGCTATGGAT TAAGCGTATG  [ 300]
AibiCR2 C......... .......... .......... .......... .......... .......... .......... .......... .......... ..........  [ 300]

AibiCR1 GCTGTGCTTA AGTTCAGTTG ATTGTAATGG TGACAGGCCA TGCTAGTTCA ATTGTCTCTT GAAGTACCGG TATCTGAAGT ACCAGGTTAT TTATTAGTCG  [ 400]
AibiCR2 .......... .......... .......... .......... .......... .......... .......... .......... .......... ..........  [ 400]
                F box                                           E box
AibiCR1 TTCCTCTCAC GTGAAATCAG CAACCCGCCG CATATAAGGC TCTACGTTAC TAGCTTCAGG ACCATTCTTT CCCCCTACAC CCTAGCACAA CTTGCTCTTT  [ 500]
AibiCR2 .......... ...G...... .......... .......... .......... .......... .......... .......... .......... ..........  [ 500]
                  D box                                                      C box
AibiCR1 TGCGCCTCTG GTTCCTCGGT CAGGGCCATG GCTCGGTTGA CTTAGCACTT GGTGCTCTTC ACAGAGTCAT TTGGTTGATG CTTGTCTGCT TCTCACCCGT  [ 600]
AibiCR2 .......... .......... .......... .......... .......... .......... ..G....... .......... .......... ..........  [ 600]

AibiCR1 GATCGCGGCA TCTGGATTGC CTGGGGCGCC TCTAGTATTT TTCTCTTCTA AACTTCTTCA GGCAGCCCTC CGGTGCACCG CGGCGCAGCC ATCGAAGACG  [ 700]
AibiCR2 .......... .......... .......... .......... .......... .......... .......... .......... .......... ..........  [ 700]
                                                                                                         BSB
AibiCR1 TGAGCATACA GACGCGTCAT CGGCCTCTTA TTAGCACTCA GGAATGACTG GATGAGACGG TTGGAGTATT TGGGGAATCA TTTTTACACT GTGCACTTTG  [ 800]
AibiCR2 .......... .......... .......... .......... .......... .......... .......... .......... .......... ..........  [ 800]
          B box                                                CSB1
AibiCR1 TTTTCCATTT GGTTGTTGGT GTGTCCACTA ACCCTAAAT ATGCTGCTAT TTGTTGAATG CTTGTTGGAC ATGATTTTAT CTCTATTTTA CTTATTTACA  [ 900]
AibiCR2 .......... .......... .......... .......... .......... .......... .......... .......... .......... ..........  [ 900]

AibiCR1 CTTCCTCTAA TTTTCTTTCA CTTTATAAAT TAAACTAGGT AACTTTCATC TAAAAATTTA ACAAGCCTTA TCAAAAATTT TTCACAAATC TTATTCTTGT  [1000]
AibiCR2 .......... .......... .......... .......... .......... .......... .......... .......... .......... ..........  [1000]
```

```
                         4 x 93 bp + 1 incomplete repeat (12 bp) in CR1
AibiCR1 ATTTTACATT ACCTTAAACC ACTGAAATTA CATTAAAAAA CATACCCTAA CACAGGGTAA CTTTTCTTAG TGTGTTATTG TATATTTACA CGTAATTATT  [1100]
AibiCR2 .......... .......... .......... .......... .......... .......... .......... .......... .......... ..........  [1100]
                         3 x 93 bp + 1 incomplete repeat (12 bp) in CR2
AibiCR1 ACCCTTTAAT ACCACTAGAA TTACATTAAA AAACATACCC TAACACAGGG TAACTTTTCT TAGTGTGTTA TTGTATATTT ACACGTAATT ATTACCCTTT  [1200]
AibiCR2 .......... .......... .......... .......... .......... .......... .......... .......... .......... ..........  [1200]

AibiCR1 AATACCACTA GAATTACATT AAAAAACATA CCCTAACACA GGGTAACTTT TCTTAGTGTG TTATTGTATA TTTACACGTA ATTATTACCC TTTAATACCA  [1300]
AibiCR2 .......... .......... .......... .......... .......... .......... .......... .......... .......... ..........  [1300]

AibiCR1 CTAGAATTAC ATTAAAAAAC ATACCCTAAC ACAGGGTAAC TTTTCTTAGT GTGTTATTGT ATATTTACAC GTAATTATTA CCCTTTAATA CCACTAGAAT  [1400]
AibiCR2 ....------ ---------- ---------- ---------- ---------- ---------- ---------- ---------- ---------- -------...  [1400]

AibiCR1 TACATTAAAT AAAACAAACA TTATTATGCT TCACGTAACT AATTTC---- ---------- ---------- ---------- ---------- ----------  [1500]
AibiCR2 .......... .......... .......... .......... ..A...CATA TCCCCCTTAC ACTAAACAGC ACTAAAATCT CAAGTAAACA ACAAACAACA  [1500]

AibiCR1 ---------- ---------- ---------- ---------- ---------- ---------- ---------- ---------- ---------- ----------  [1600]
AibiCR2 AACAAACAAC AAACAAACAA CAAACAACAA ACAAACAACA AACAACAAAC AACAAACAAA CAACAAACAA ACAACAAACA ACAAACAAAC AACAAACAAA  [1600]

AibiCR1 ---------- ---------- ---------- ---------- ---------- ---------- ---------- ---------- ---------- ----------  [1700]
AibiCR2 CAACAAACAA ACAACAAACA ACAAACAACA AACAACAAAC AAACAAACAA CAAACAACAA ACAAACAAAC AACAAACAAC AAACAACAAA CAAACAAACA  [1700]

AibiCR1 ---------- ---------- ---------- ---------- ---------- ---------- ---------- --- [1773]
AibiCR2 ACAAACAACA AACAAACAAA CAACAAACAA CAAACAAACA AACAACAAAC AACAAACAAC AAACGATCAC ACT [1773]
```
