# Supplementary material for: The complete mitochondrial genomes of sixteen ardeid birds revealing the evolutionary process of the gene rearrangements
Source: BMC Genomics. 2014 Jul 8;15(1):573. doi: 10.1186/1471-2164-15-573 (PMC4111848; doi:10.1186/1471-2164-15-573)
Supplement: Supplementary file 4 — Additional file 4: Pair-wise alignments of subsections between the sizable no coding sections and corresponding functional genes within individuals of five species. NC1: no coding section between tRNAThr and tRNAGlu1 or CR1; NC2: no coding section between CR1 and tRNAPro. Numerals in brackets indicate the positions of the aligned sequences. Vertical lines indicate boundary of the gene. Dots indicate identity of nucleotides to the reference sequence; dashes indicate gaps. (PDF 31 KB) [file 12864_2014_6274_MOESM4_ESM.pdf]

A.

68.8% identity in 141 nt overlap tRNA^{Pro} | |ND6
A. cinerea tRNA^{Pro}-ND6 CAACTCCCAAAGCTGGCATTTTATACTAAACTACTTTC-TGAACTCCGTACCAA-CCTAACTGCCCGAAGAGCCCCACGA
A. cinerea NC1 (26-157) ....C.........--...CC....---...C...CA.C.A....A.AC.T...A..C...CA...-...TA...A.A..

A. cinerea tRNA^{Pro}-ND6 GATAACCCCCGCACTAACTCTAACACAACAAACAAAGTTAACAGTAACCCTCAACCTCCCA
A. cinerea NC1 (26-157) A....A..T.A.......C--......G.......G.C..C..-.C.T..C..C...A.A.

85.7% identity in 42 nt overlap ND6|
A. cinerea ND6 CACAAAGCATAGCCCTAAAAAAATTACAAAGTAAGTCATAGC
A. cinerea NC1 (176-217) .T...CT.T..A.......................C......

72.3% identity in 148 nt overlap
A. cinerea Cytb-tRNA^{Thr} AGCCTCCATTACCTACTTCACAATCCTCCTAATCCTATTCC-CCATTACTGGAGCCCTAGAAAACAAAATACTAAATTAC
A. cinerea NC2 (13-154) .T..A..TCCG..C.....-..--.....CC....CTC...T...C.......A...-..............C...CC..
Cytb| |tRNA^{Thr}
A. cinerea Cytb-tRNA^{Thr} TAAACCACTCTAATAGTTTATTAAAAACATTGGTCTTGTAAACCAAAGAATGAAGACTATACCCCTTC
A. cinerea NC2 (13-154) C...T....--..C.A....C...G.G....AAC.C..CT..............A....C....A.C.

B.

63.0% identity in 208 nt overlap
A. purpurea ND6 CACAAAAACTACTAACATTCCACCCAGATACACCAGAAACAGCACCAGCGAAATAAAAGAAACCCCCAAACTCAACAACC
A. purpurea NC1 (25-218) ......CT.C..ATCA.GC....T.CC.G..G..C-...G.A.C.ACA.A.C.G.C..C.CC..A...C.AAA.TA..A.

A. purpurea ND6 ACCCACACCCTACAACAGATGCCAGAACTAAACCAACAACC--CCATAATAGGGTGACGGGTTCGAAGCAACCGCTAGTC
A. purpurea NC1 (25-218) .T.-.......-....---.T....C..CTT..AG..CT..AA..GA..C.-----..A.AC.....A.....T.C..CT
ND6 |
A. purpurea ND6 CACCTAACACAAAACATAACCCTAAAAAAACTACAAAATAAGTCATAG
A. purpurea NC1 (25-218) ....C........-........C.....--.............C..C..

68.0% identity in 153 nt overlap
A. purpurea Cytb-tRNA^{Thr} AACTA-GCCTCCGTCACCTACTTCATAATCCTCCTGATCCTATTCCCCATTACTGGAGCCCTAGAAAACAAAATACTAAA
A. purpurea NC2 (12-152) ..A..T....T.A.....C.....CC..C...A..----------....C...C.A.......-...........T...C
Cytb | | tRNA^{Thr}
A. purpurea Cytb-tRNA^{Thr} CTACTAAATTACTCTAATAGTTTACTAAAAACATTGGTCTTGTAA-ACCAAAGGATGAAGACTATACCCCTTC
A. purpurea NC2 (12-152) ....C...CC..C....C.AC.........-....AAC.C...T.G......A..AC..CCAC.CT....A..

C.

76.3% identity in 76 nt overlap tRNA^{Pro} |
A. modesta tRNA^{Pro} AGAAAGAGAGAGCTCAAACCTCCATCTCCAACTCCCAAAGCTGGTATTTTATACT--AAACTACTTTCTGAACTCC
A. modesta NC1 (39-114) ..GG.A................T.........C.........A...C.CC.....TG....CG..CC..T.C.C..

68.5% identity in 143 nt overlap
A. modesta Cytb-tRNA^{Thr} AACTAGCCTCCATCACCTACTTCATAATCCTACTAATCCT-AT-TC-CCCATCACTGGAGCCCTAGAAAATAAAATACTA
A. modesta NC2 (14-145) ...ACA.......TC..-..C...C.....--.C.GC...T..G..T....C..A.A......C.....----....AA.
Cytb | | tRNA^{Thr}
A. modesta Cytb-tRNA^{Thr} AACTACTAAACCACTCTAATAGTTTACCAAAAACATTGGTCTTGTAGACCAAAGAATGAAGGC
A. modesta NC2 (14-145) C.....----....C....C..C....T.........A.C.C.AC.AG......C..A..A..

D.

63.6% identity in 231 nt overlap
|tRNA^{Pro} tRNA^{Pro}|
A. intermedia tRNA^{Pro}-ND6 ACTCAGAAAGAGAGAGCTCAAACCTCCATCTC----CAACTCCCAAAGCTGGCTATTTTACACTAAACTACTTTCTGAAC
A. intermedia NC1 (18-243) .G...A.........A.CT.......T.....ATCT...........A....-.....C..........T..C..CA.T.
|ND6
A. intermedia tRNA^{Pro}-ND6 TCCACACTAAACCTAACTGCCCGAAGAGCCCCACGAGATAACCCCCGCACTAACTCTAACACGACAAACAGAG--TTAAC
A. intermedia NC1 (18-243) .T-...TC........TCA..TA....A.T....A.A.C.......A.C.AT..AAGC.GCAC....C..TGTCC.C..G

A. intermedia tRNA^{Pro}-ND6 AACAGCCC--CCAACCTCCTACTAAA-----AACATCCCCACTCCATGCGAATAAAACATAGCTACACCTC
A. intermedia NC1 (18-243) ....A..TAA.A..GAG.A....T..CCCCC..T.A..T...AGGCCT.C.T...C...---..T..A...

63.6% identity in 129 nt overlap Cytb | | tRNA^{Thr}
A. intermedia Cytb-tRNA^{Thr} TTCCCCATCACTGGAGCCTTAGAAAATAAAATACTAAACTACTAAACCACTCTAATAGTTTACTAAAAACATTGGTCTTG
A. intermedia NC2 (16-137) .A....T...TCCCCA..CC.C..CTCC.G.A.AC.....--.G......C.C..C.C..C.....G.CA.AC---.C.A
tRNA^{Thr}|
A. intermedia Cytb-tRNA^{Thr} TAAACCAAAGAATGAAGACTATACCCCTTCTTAGAGT-TAGACACCCAC
A. intermedia NC2 (16-137) C..........G....A..C.-......-......ACC..A........

E.

60.5% identity in 162 nt overlap
A. ibis ND6 (324-482) AAACTACTAACATTCCACCCAAGTACACCAAAAACAACACCAATGAGATAAAAGAAACACCCAAGCTCAACAACCATCCA
A. ibis NC1 (2-156) .....T.C.C.CAG...T....AAG.C...CTC.ACC...AC.GC...C...TC.TTA...-.CT.......-...G...

A. ibis ND6 (324-482) CACCCA-AC--AACAGACGCCAAAACCAAACCAACAACCCCATAGTAAGGTGACGGATTCGAAGCAACTGCCAACCCACCTA
A. ibis NC1 (2-156) T.A...C..TG...TT..A.--.CT...G.T.C......--...AC.GAA.CG..A.GCT..-.T......T........C.

62.6% identity in 131 nt overlap Cytb | | tRNA^{Thr}
A. ibis Cytb-tRNA^{Thr} CTACTTTACGATCCTCCTAGTCCTATTCCCTATCACTGGGGCCTTAGAGAACAAGATACTAAACTACTAACCACTCTAAT
A. ibis NC2 (33-146) ......-..--...-..C.AC....---........A---A.TCC.A.A.....AGC..--...C.......T..C..C..

A. ibis Cytb-tRNA^{Thr} -AGTTTATTAAAAACATTGGTCTTGTAAACCAAAGAATGAAGACTATACCC
A. ibis NC2 (33-146) C.A..C.CC........CAAA.----G.......-...A..A..C.CG...
